# *In silico* design of novel indolinone-based anticancer agents and proposal of synthetic routes

**DOI:** 10.3389/fbinf.2026.1964610

**Published:** 2026-10-14

**Authors:** Mohammed Er-rajy, Mohamed El Fadili, Hanane Etabti, Arzine Aziz, Fatimazohra Lenda, Somdutt Mujwar, Samir Chtita, Abdelouahid Samadi, Menana Elhallaoui

**Affiliations:** 1 LIMAS Laboratory, Faculty of Sciences Dhar El Mahraz, Sidi Mohamed Ben Abdellah University, Fez, Morocco; 2 Engineering Laboratory of Organometallic, Molecular Materials, and Environment (LIMOME). Dhar El Mahraz, Sidi Mohamed Ben Abdellah University, Fez, Morocco; 3 Physical-Chemical Laboratory of Materials, Natural Substances and the Environment (LAMSE), Department of Chemistry, Faculty of Sciences, Techniques of Tangier-University Abdelmalek Essaadi, Tangier, Morocco; 4 Chitkara College of Pharmacy, Chitkara University, Rajpura, Punjab, India; 5 Laboratory of Analytical and Molecular Chemistry, Faculty of Sciences Ben M’Sik, Hassan II University of Casablanca, Casablanca, Morocco; 6 Department of Chemistry, College of Science, United Arab Emirates University, Al Ain, United Arab Emirates

**Keywords:** anticancer agents, indolinone, molecular docking, molecular dynamics, POM analysis, QSAR method, synthetic routes

## Abstract

The RAF–MEK–ERK signaling pathway plays a central role in cancer development and progression and represents an important target for anticancer drug discovery. Simultaneous targeting of components of this pathway may therefore represent a promising strategy for cancer treatment. A quantitative structure-activity relationship (QSAR) model was developed using multiple linear regression (MLR). The QSAR model showed satisfactory predictive performance based on complementary internal and external validation metrics. The selected molecular descriptors were used to guide the structural modification of substituents, resulting in higher predicted activity. Accordingly, ten new molecules were designed through strategic substituent modifications. Synthetic routes for these compounds were also proposed based on reported literature procedures. Molecular docking, molecular dynamics (MD) simulations, and Petra, Osiris, and Molinspiration (POM) analyses supported the prioritization of Pred 9 and Pred 10 as computationally designed candidates for further investigation. In silico absorption, distribution, metabolism, excretion, and toxicity (ADMET) predictions indicated that Pred 9 and Pred 10 may exhibit favorable pharmacokinetic properties. Overall, the results support the consideration of these compounds for experimental synthesis and biological evaluation. However, Pred 9 and Pred 10 have not yet been synthesized or experimentally evaluated for MEK1 inhibition or anticancer activity.

## 1 Introduction

Cancer encompasses a heterogeneous group of diseases characterized by uncontrolled cell growth and proliferation, resulting from the accumulation of genetic and epigenetic alterations. Lung cancer is particularly deadly because it is often asymptomatic in its early stages (Petty, 2001). In 2022, lung cancer was the leading cause of cancer-related death worldwide, with about 1.8 million deaths, accounting for 18.7% of all cancer deaths (Zhang et al., 2025). The high mortality associated with this disease, together with the emergence of multiple mechanisms of treatment resistance, highlights the continuing need for more effective therapeutic strategies and the development of new anticancer agents (Newman-Toker et al., 2021).

The RAF-MEK-ERK signaling pathway, also called the MAPK pathway, helps regulate cell growth, differentiation, and survival (McCubrey et al., 2007). It is frequently dysregulated in cancers, including lung cancer. Mutations in key proteins such as RAS and BRAF can lead to constitutive activation of this pathway, thereby promoting uncontrolled tumor growth and potentially limiting the response to standard therapy (Ryan and Corcoran, 2018). Inhibitors of the RAF–MEK–ERK signaling pathway are now considered useful treatment options for lung cancer (Han et al., 2021). These inhibitors can reduce tumor growth and trigger apoptosis by disrupting abnormal signaling in this pathway. RAF-targeted inhibitors such as vemurafenib and dabrafenib act primarily on mutant BRAF, whereas MEK inhibitors, such as trametinib and selumetinib, inhibit MEK-mediated phosphorylation and activation of ERK1/2, thereby suppressing downstream MAPK signaling. ERK inhibitors under development aim to block the final step of the pathway and may help patients who become resistant to inhibitors that act upstream. Clinical studies have indicated potential benefits of RAF–MEK–ERK pathway inhibitors, especially in tumors with KRAS or BRAF mutations, but resistance remains a major clinical problem (Yap et al., 2011). Lung cancer cells may activate alternative signaling pathways, so treatment may require drug combinations to potentially reduce the risk of relapse. Recent studies are examining whether combining these inhibitors with other targeted drugs, immunotherapy, or standard chemotherapy can improve patient outcomes (Vanneman and Dranoff, 2012). Although several components of the RAF-MEK-ERK cascade can be therapeutically targeted, the present study specifically focuses on MEK1 as the molecular target. The broader discussion of the RAF-MEK-ERK pathway is included to establish the biological context and therapeutic relevance of MEK1 inhibition.

Among the components of the RAF-MEK-ERK signaling cascade, MEK1 represents a particularly relevant molecular target because it acts as a central signaling component between RAF and ERK and directly regulates the phosphorylation and activation of ERK. Aberrant activation of MEK1/ERK signaling contributes to cancer cell proliferation, survival, and disease progression (Samatar and Poulikakos, 2014). Therefore, MEK1 inhibition may represent a strategy to interfere with abnormal MAPK signaling downstream of oncogenic alterations occurring in upstream components of the pathway (Caunt et al., 2015).

Indolinone derivatives have attracted considerable attention as privileged scaffolds in the development of kinase-targeting compounds because their structural framework can be readily modified to explore interactions with kinase-binding sites and to optimize biological activity (Prakash and Raja, 2012). In particular, structural modifications of the indolinone scaffold may influence steric, electronic, hydrophobic, and other physicochemical properties that are relevant to ligand–target interactions. These characteristics make indolinone derivatives an appropriate chemical series for investigating structure–activity relationships and identifying structural features potentially associated with MEK1 inhibitory activity.

Despite the therapeutic relevance of MEK1 and the continued interest in MEK1-targeted drug discovery, the relationship between the molecular structure of indolinone derivatives and their biological activity remains insufficiently understood. In particular, the structural and physicochemical features that contribute to variations in activity within this chemical series require further investigation (Tropsha, 2010). Therefore, computational approaches capable of identifying relevant molecular descriptors and using these descriptors to guide the rational design of new candidate compounds may provide valuable insights into the structure–activity relationships of these derivatives (Cherkasov et al., 2014).

Building on our previous computational studies, the present work focuses on a distinct series of indolinone derivatives and differs from our earlier QSAR investigations in both its chemical dataset and its integrated computational strategy. The novelty of this study lies in combining descriptor-based QSAR modeling with QSAR-guided molecular design and complementary molecular modeling approaches, including molecular docking targeting MEK1, MD simulations, POM analyses, and *in silico* ADMET predictions, to prioritize newly designed computational candidates for further experimental investigation (Ferreira and Andricopulo, 2018; Elhenawy et al., 2019; Aloui et al., 2025; Er-Rajy et al., 2025; Berrahou et al., 2026).

Based on the hypothesis that specific structural and physicochemical descriptors of indolinone derivatives are associated with their reported biological activity, the objective of this study was to develop and validate a QSAR model and to use the identified structure–activity relationships to guide the design and prioritization of new computational candidates based on their predicted activity (Er-rajy et al., 2022a; Er-Rajy et al., 2025). The most promising computational candidates were subsequently evaluated using molecular docking against MEK1, MD simulations, POM analysis, and *in silico* ADMET predictions (Er-rajy et al., 2023e; Er-Rajy et al., 2024; Arzine et al., 2026a). The newly designed compounds are computational candidates only and have not yet been synthesized or experimentally evaluated for MEK1 inhibition or anticancer activity. Therefore, the present findings should be regarded as predictive and hypothesis-generating and are intended to support the prioritization of candidates for future experimental synthesis and biological evaluation.

## 2 Materials and methods

### 2.1 Experimental dataset

This study examines a dataset on the anticancer activity of 33 hybrid 3-substituted 5-(phenylamino) indolone derivatives, which were divided into three groups (Yu et al., 2019). The first group comprises six molecules (1–6), the second group comprises four compounds (7–10), and the third group comprises twenty-three molecules (11–33), as shown in Scheme 1. In addition, Supplementary Table S1 presents the data of the studied compounds along with their IC_50_ values.

**SCHEME 1:**
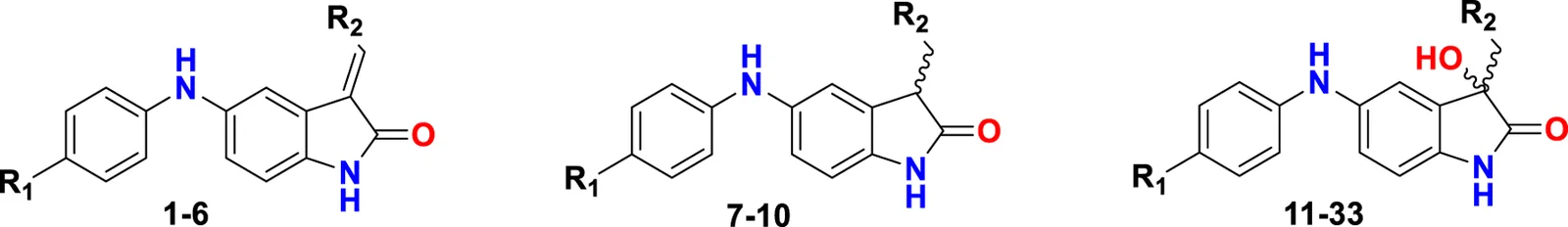
Different groups of indolinone derivatives.

**SCHEME 2:**
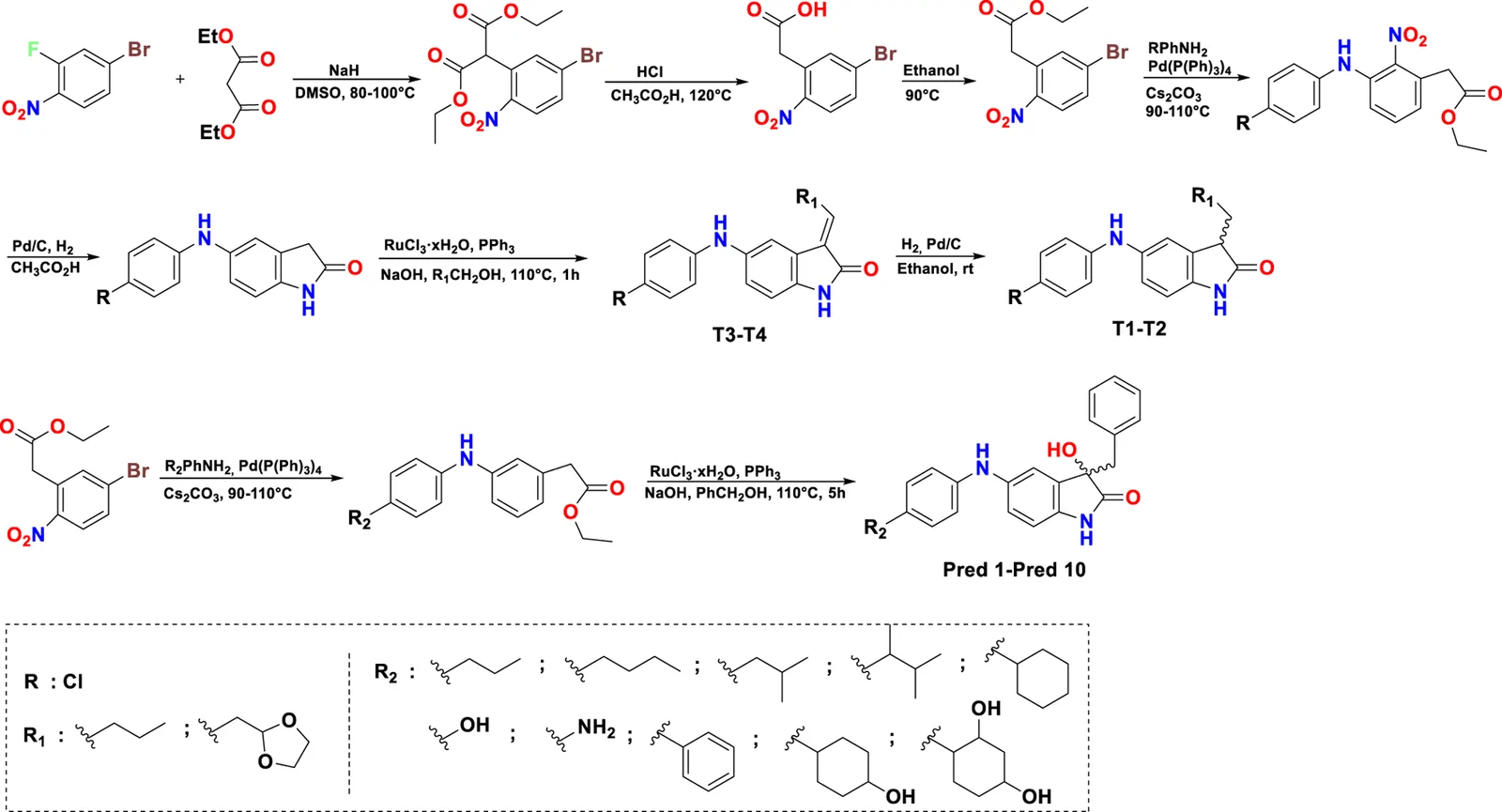
Hypothetical synthetic route proposed for the preparation of Pred 9 and Pred 10 based on related literature-reported synthetic procedures.

### 2.2 Descriptor analysis and QSAR model development

To build the QSAR model, we used descriptors that capture physicochemical, topological, and electronic properties. We calculated the descriptors using the Gaussian 03W software package (Tomberg, 2013). Physicochemical and topological descriptors were calculated using Chem Bio Office 3D software (Zielesny, 2005). After descriptor calculation, the compounds were randomly split into two sets: 27 compounds in a training set for model development and 6 compounds in a test set to evaluate the predictive performance of the final model. Recent advances in technology have made it possible to compute many different molecular properties. The risk of spurious correlations increases when a multiple linear regression (MLR) model contains many descriptors relative to the number of compounds used to develop the model (Roy and Pratim Roy, 2009). Although different optimization methods can produce models that match the experimental data, overfitting can still be a concern. Overfitting can limit a model’s ability to generalize; therefore, its predictive performance should be assessed using separate validation data before drawing conclusions.

### 2.3 Dataset division

The dataset consisted of 33 compounds with experimentally reported IC_50_ values, which were converted to pIC_50_ values for QSAR modeling. The compounds were randomly divided into a training set and an external test set using an approximate 80:20 ratio (Tropsha, 2010). Accordingly, 27 compounds were randomly assigned to the training set for the development of the QSAR model, whereas the remaining 6 compounds were retained as an external test set for the independent evaluation of the model’s predictive performance. The test compounds were not used during model development.

### 2.4 Descriptor selection

Molecular descriptors were calculated for all compounds and subsequently screened to identify descriptors relevant to the biological activity. The final QSAR model was developed using six selected descriptors, namely Bend, Stretch-Bend, 1,4 VDW, NHA, TC, and TVC. These descriptors were used to develop the MLR model.

### 2.5 Y-randomization

The robustness of the developed QSAR model was evaluated using a Y-randomization (Y-scrambling) test (Tropsha et al., 2003). The dependent variable (pIC_50_) was randomly permuted 500 times, while the original matrix of the six molecular descriptors (Bend, Stretch-Bend, 1,4 VDW, NHA, TC, and TVC) was kept unchanged. For each randomized dataset, a new multiple linear regression model was generated using the same descriptors and the same modeling procedure as those applied to the original QSAR model. The statistical performance of each randomized model was evaluated using the coefficient of determination (R^2^) and the leave-one-out cross-validation coefficient (Q^2^LOO) (Tropsha et al., 2003; Er-Rajy et al., 2025). The resulting statistical parameters were then compared with those of the original model. A substantial decrease in the R^2^ and Q^2^LOO values of the randomized models compared with those of original model was considered evidence that the developed QSAR relationship was not due to chance correlation.

### 2.6 Assessment of multicollinearity

Potential multicollinearity among the descriptors included in the QSAR model was assessed using variance inflation factor (VIF) analysis (O’Brien, 2007). The VIF values were calculated for that descriptor by regressing each descriptor against the remaining descriptors according to the following equation:

$$VIF=\frac{1}{1-R^{2}}$$

where R^2^ is obtained from the regression of a given descriptor against all other descriptors. The VIF values were subsequently used to evaluate the degree of multicollinearity among the selected molecular descriptors.

### 2.7 ADMET properties and drug-likeness

A clear understanding of ADMET properties is essential for assessing whether new drug candidates are suitable for further development during the drug discovery process (Kowalska et al., 2018). A compound’s drug-likeness is also important because it provides an indication of its potential suitability for development as an orally administered drug. To this end, we used the SwissADME and pkCSM web servers to perform *in silico* analyses of M31 and the two proposed new compounds, Pred 9 and Pred 10 (Daina et al., 2017). We assessed lipophilicity, aqueous solubility, saturation, molecular flexibility, and a range of pharmacokinetic properties that influence drug bioavailability. Drug-likeness was assessed according to established drug-likeness criteria (Matin et al., 2022).

### 2.8 Molecular docking and MD simulations

#### 2.8.1 Protein and ligand structure preparation

The three-dimensional structure of the target protein was obtained from the Protein Data Bank (PDB) under PDB accession code 3EQG (Fischmann et al., 2009). The crystal structure was determined by X-ray crystallography at a resolution of 2.50 Å. The protein structure was prepared for subsequent molecular docking and MD simulations by removing non-essential crystallographic components, when required.

The co-crystallized ligand present in the crystal structure was identified as Mirdametinib (PD-0325901) and was used as a reference ligand for validation of the molecular docking protocol. The experimentally observed binding site of this ligand was used to define the docking region.

#### 2.8.2 Molecular docking and MD simulations protocol

Prior to the molecular docking study, the two most active molecules were drawn using ChemDraw 16.0, and their geometries were optimized using the MM2 force field (Allinger, 1977; Er-rajy et al., 2022a). For the lung cancer study, we utilized the MEK1 protein structure obtained from the Protein Data Bank (PDB ID: 3EQG) (Iiams et al., 2011).

The docking grid was centered on the binding site occupied by the co-crystallized ligand. The grid center was defined at X = −4.762 Å, Y = 61.761 Å, and Z = 34.127 Å. We performed molecular docking using AutoDock 4 (Morris et al., 2008). Docking studies were conducted using the Lamarckian genetic algorithm to find the protein-ligand complex with the lowest predicted binding free energy. For the molecular docking study, the search grid was set to 65 × 65 × 65 points along the X, Y, and Z axes. For the molecular docking studies, we generated 100 candidate solutions for each case using a population size of 350. The grid dimensions were selected to adequately encompass the binding cavity and provide sufficient space for conformational sampling of the investigated ligands.

To examine ligand–protein interactions, we removed water molecules, repaired missing side-chain residues, and merged nonpolar hydrogens using Discovery Studio 2020 (Pinzi and Rastelli, 2019). Following the molecular docking study, MD simulations were conducted to assess the stability of the obtained complexes (Parihar et al., 2022; Er-rajy et al., 2023f). Using the Desmond program within the Schrödinger Suite, MD simulations were run for 100 nanoseconds (ns) with the OPLS3e force field (Miao et al., 2019; Jawarkar et al., 2025). The Pred 9–3EQG and Pred 10–3EQG complexes were simulated independently under the same MD simulation conditions for 100 ns each.

## 3 Results

### 3.1 Data collection and dataset preparation

The QSAR model was developed using the experimentally measured anticancer activity data for the 33 selected compounds and their corresponding descriptor values, as listed in Supplementary Table S2. The dataset was then randomly divided into a training set comprising 27 compounds and a test set comprising 6 compounds. MLR analysis was subsequently performed iteratively to refine the model by retaining statistically significant descriptors and eliminating variables that contributed minimally to the model performance, as assessed by R^2^.

### 3.2 MLR method

The MLR method is commonly used in QSAR studies because it is simple, easy to interpret, and yields reproducible results. We assessed the reliability of the proposed model using the MLR approach with two metrics: R^2^ and the root-mean-square error (RMSE) (ER-RAJY et al., 2022b; Er-rajy et al., 2023f). The QSAR model developed using the MLR method is given by Equation 1.

$$\begin{array}{ll} & {\text{pIC}}_{50}=1,67403-0,04656^{*}\left(\text{Bend}\right)+2,38681^{*}\left(\text{Stretch}-\text{Bend}\right)\\ & \;+0,19034^{*}\left(1,4\text{ VDW}\right)+0,10543^{*}\left(\text{NHA}\right)\\ & \;+2631^{*}\left(\text{TC}\right)-76125^{*}\left(\text{TVC}\right) \end{array}$$ (1)

$R_{Training}^{2}$ = 0.741; R^2^ adjust = 0.663; RMSE = 0.144.

Additional statistical parameters obtained from the internal and external validation of the developed QSAR model are presented in Table 1. The coefficient normalization diagram is shown in Figure 1.

**TABLE 1:** Some external validation parameters of our model QSAR.

Parameter	Threshold	Modelscore
$Q_{\text{training}}^{2}$	$Q_{\text{training}\;}^{2}$ > 0.5	0.700
$R_{Training}^{2}$	$R_{Training}^{2}$ > 0.6	0.741
$R_{Test}^{2}$	$R_{Test}^{2}$ > 0.6	0.726
$r_{0}^{2}$		0.65
$r_{0}^{\prime 2}$		0.74
|r_0_^2^- r’_0_^2^|	Less than 0.3	0.09
k	Between 0.85 and 1.15	1.000
$k^{\prime }$	Between 0.85 and 1.15	0.999

**FIGURE 1:**
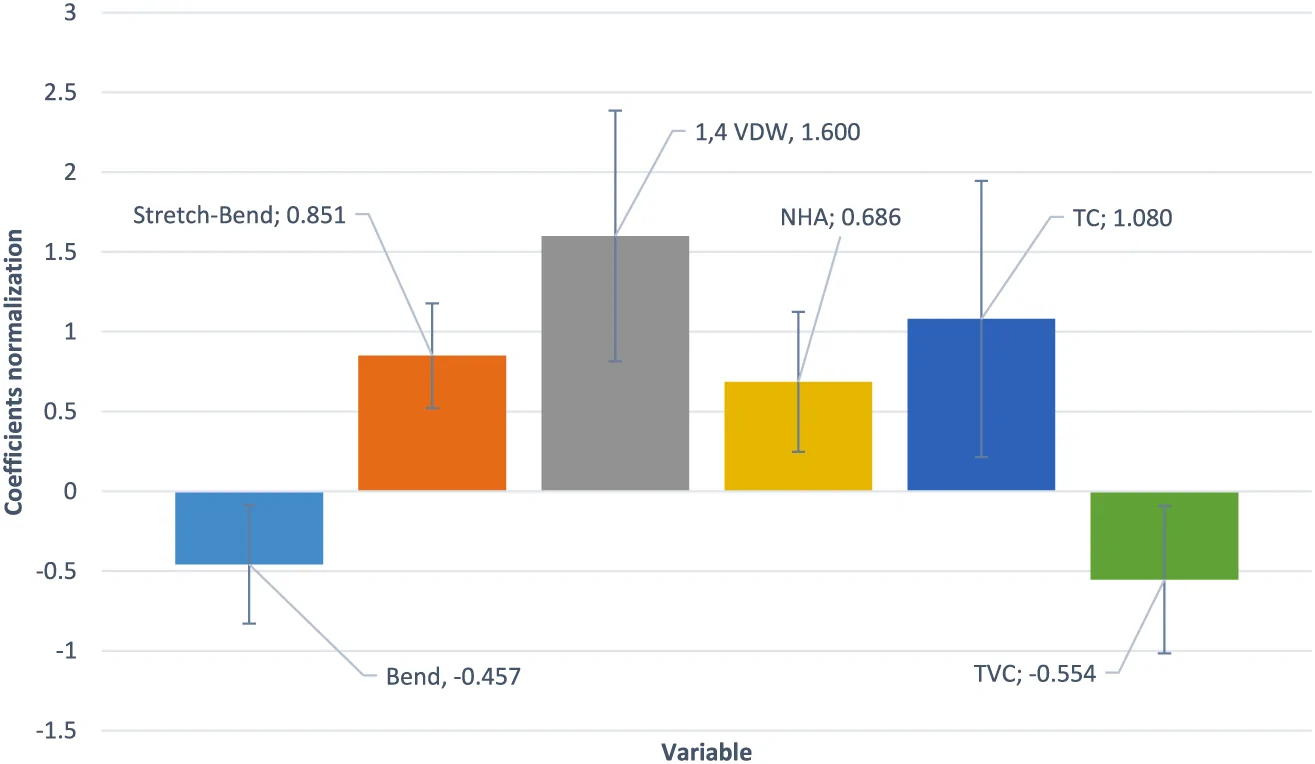
Coefficients normalization scheme.

Figure 2 therefore provides a visual representation of the agreement between the experimental and predicted activities for both the training and external test sets.

**FIGURE 2:**
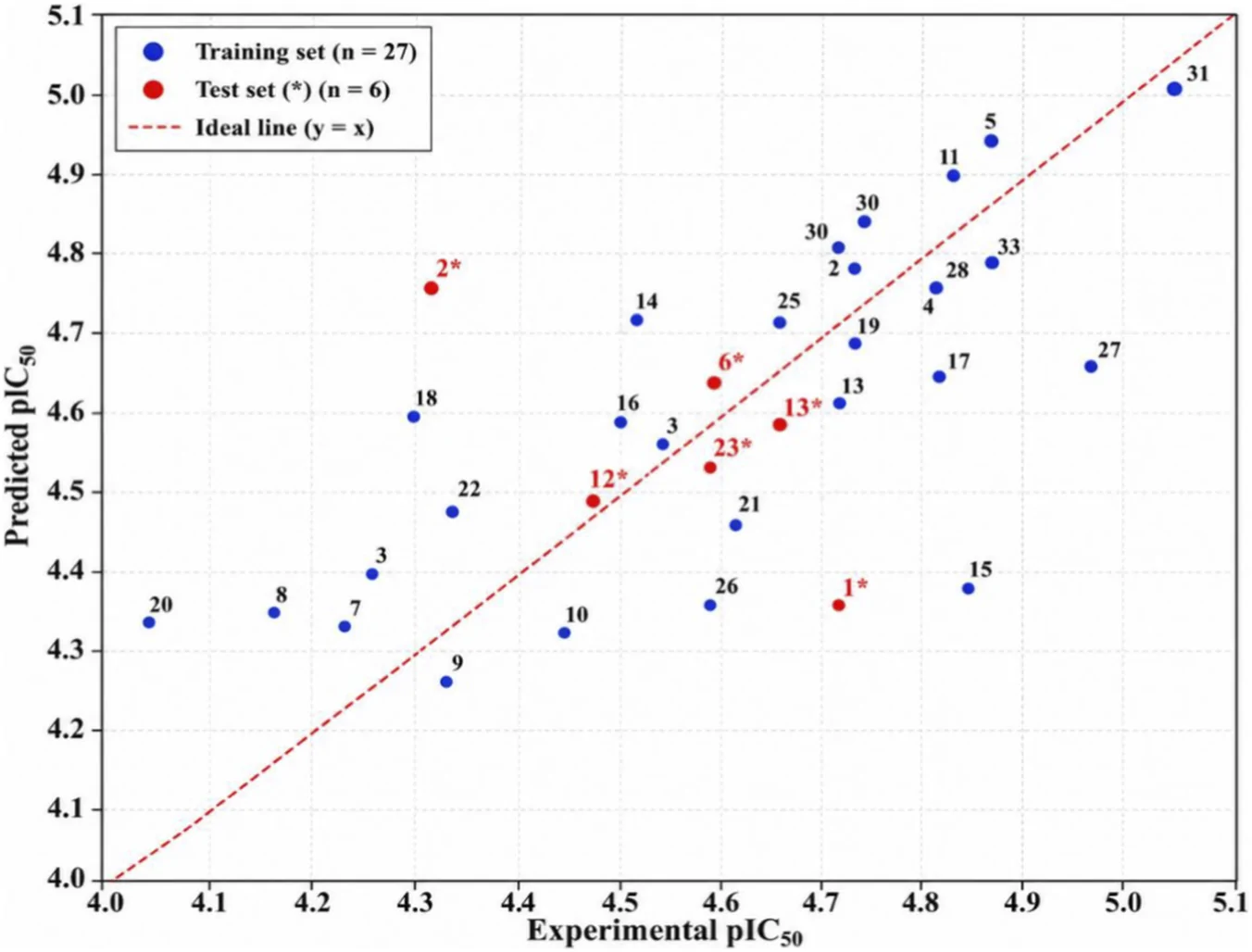
Experimental versus predicted pIC_50_ values for the studied compounds.

### 3.3 External validation

This approach is based on the criteria established by Golbraikh and Tropsha (Tropsha, 2010). Table 1 lists some of the criteria suggested for the external validation of MLR models.

### 3.4 Y-randomization

A Y-Randomization (Y-scrambling) test was performed using the dataset comprising 27 compounds and six molecular descriptors (Bend, Stretch-Bend, 1,4 VDW, NHA, TC, and TVC). The dependent variable (pIC_50_) was randomly permuted 500 times, while keeping the descriptor matrix unchanged. A summary of the Y-randomization results is presented in Table 2, while the complete results obtained from all 500 randomization runs are provided in Supplementary Table S3.

**TABLE 2:** Y-randomization results of the developed QSAR model.

Parameters	Original model	Y-randomized models
Number of compounds	27	500 randomizations
R^2^	0.8739	Mean = 0.1871
Maximum R^2^	—	0.6804
Q^2^LOO	0.7736	Mean = −0.3801
Maximum Q^2^LOO	—	0.5193

The robustness of the developed QSAR model was evaluated using a Y-randomization test. The dependent variable (pIC_50_) was randomly permuted 500 times, while the original matrix of the six molecular descriptors (Bend, Stretch-Bend, 1,4 VDW, NHA, TC, and TVC) was kept unchanged. For each randomized dataset, a new multiple linear regression model was generated using the same descriptors and the modeling procedure as those applied to the original QSAR model. The statistical performance of each randomized model was evaluated using the R^2^ and Q^2^LOO. The resulting statistical parameters were then compared with those of the original model. A substantial decrease in the R^2^ and Q^2^LOO values of the randomized models compared with those of the original model was considered evidence against the possibility of chance correlation.

### 3.5 Multicollinearity analysis

Potential multicollinearity among the six descriptors included in the QSAR model was assessed using VIF analysis. The calculated VIF values ranged from 1.98 to 5.12. Bend, Stretch-Bend, 1,4 VDW, NHA, and TVC exhibited VIF values below 5, whereas TC showed a marginally higher VIF value of 5.12. Importantly, all VIF values remained below the commonly used threshold of 10, indicating the absence of severe multicollinearity among the descriptors included in the model. Therefore, the selected descriptors were considered acceptable for inclusion in the QSAR model. The results of the multicollinearity analysis are presented in Table 3.

**TABLE 3:** VIF analysis of the molecular descriptors included in the developed QSAR model.

Descriptor	VIF
Bend	2.23800444
Stretch-bend	1.97959119
1,4 VDW	4.55575738
NHA	3.33187018
TC	5.11563494
TVC	3.87311163

### 3.6 Design of new compounds

#### 3.6.1 Molecular design strategy

The design of the new molecules was guided by the structural features of the compounds included in the original dataset and by the structure–activity relationships identified from the developed QSAR model. Structural modifications were introduced to generate new analogues while preserving the principal molecular features associated with the modeled biological activity. Particular attention was given to maintaining the descriptor values within ranges represented in the original dataset and to exploring structural modifications expected to influence the descriptors included in the final QSAR equation.

Based on this strategy, ten new compounds, designated Pred 1 to Pred 10, were designed and subsequently evaluated using the developed MLR-QSAR model. Their predicted pIC_50_ values were calculated using the QSAR equation, and the structures and corresponding predicted activities of the newly designed compounds are presented in Table 4. The compounds showing the highest predicted pIC_50_ values were subsequently prioritized for complementary *in silico* analyses.

**TABLE 4:** Structures of the newly designed compounds and their QSAR-predicted pIC_50_ values.

Molecules ID	Structure	Predicted pIC_50_ by MLR model
Pred 1	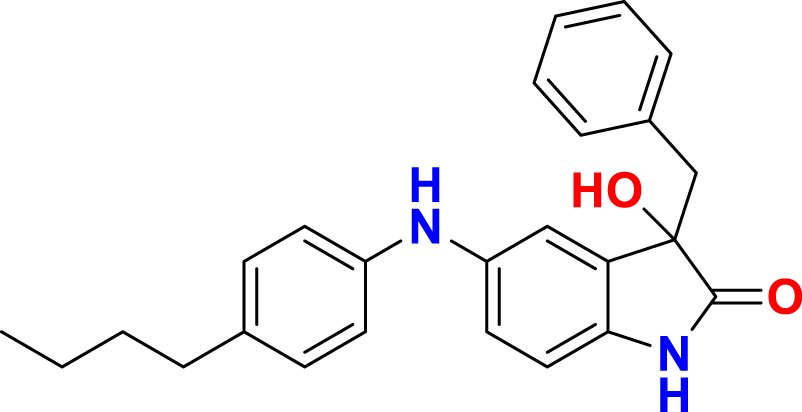	5.221
Pred 2	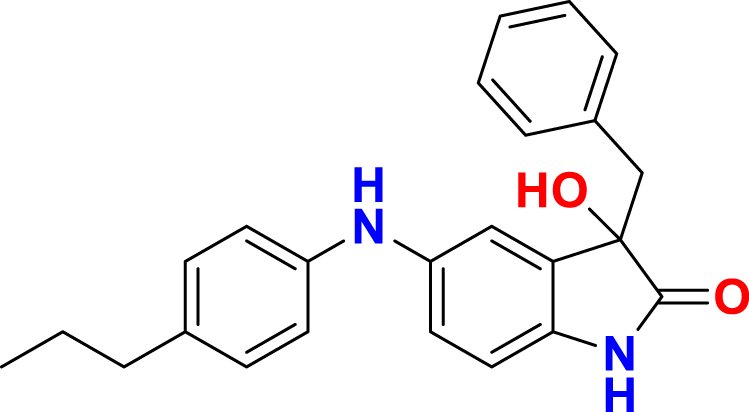	5.068
Pred 3	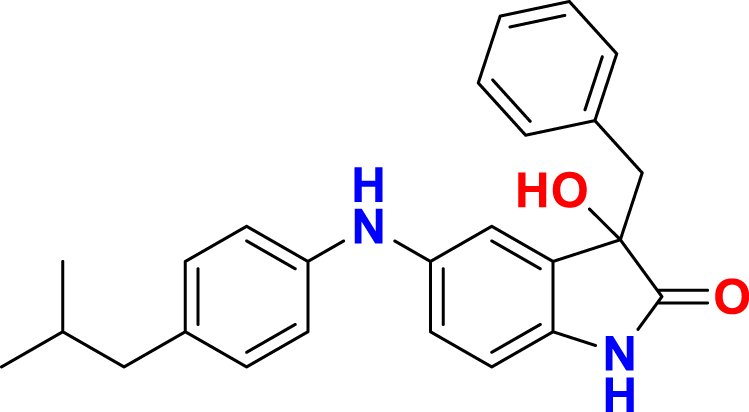	5.229
Pred 4	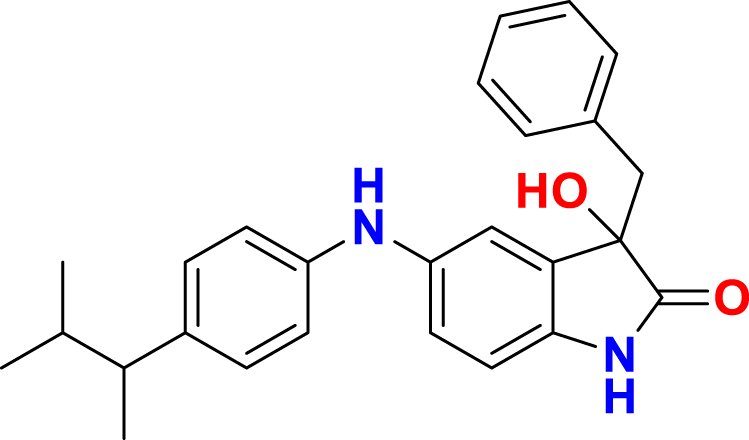	5.539
Pred 5	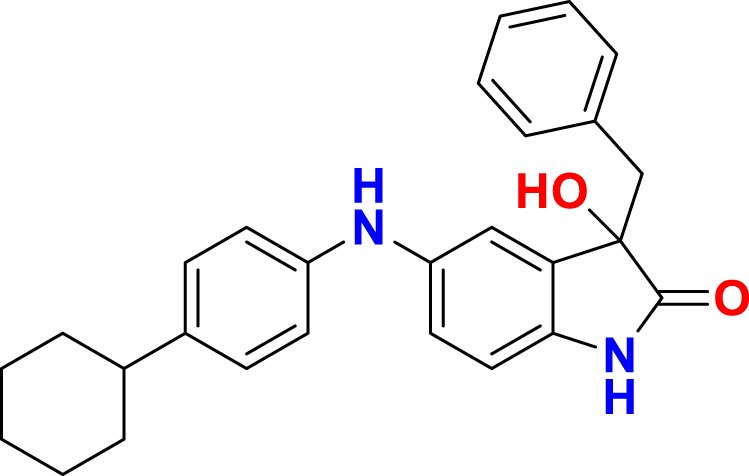	5.811
Pred 6	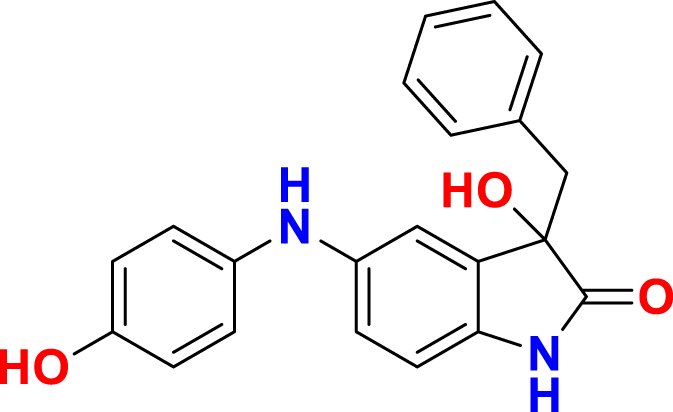	4.395
Pred 7	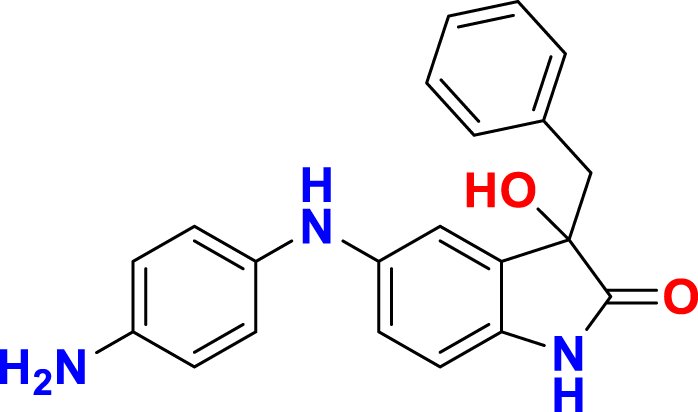	4.624
Pred 8	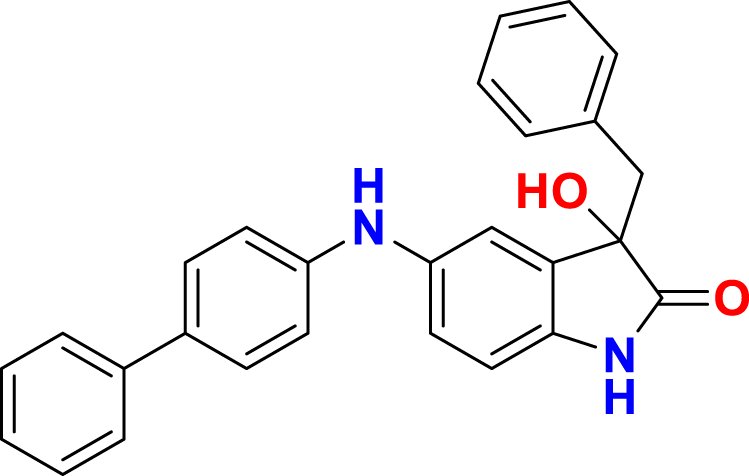	5.741
Pred 9	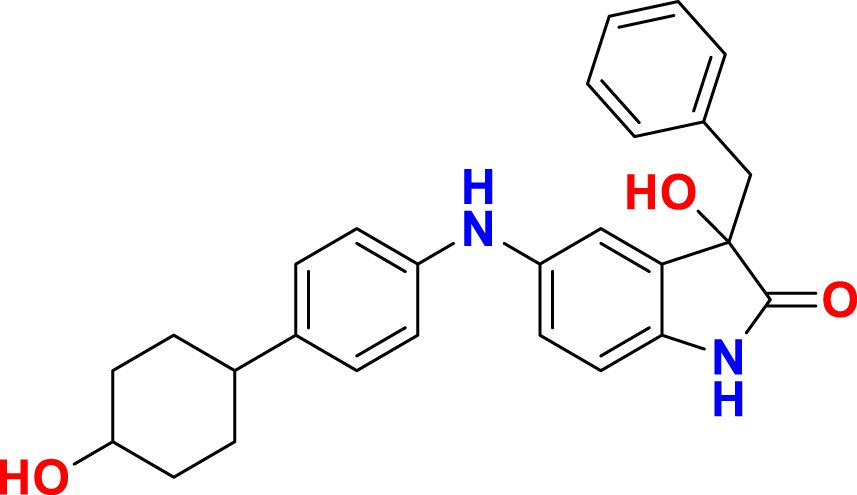	6.046
Pred 10	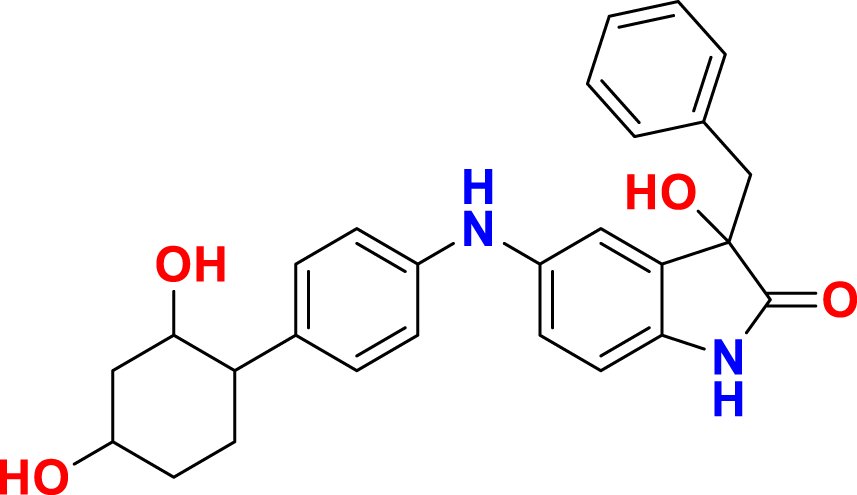	6.306

#### 3.6.2 Proposed synthetic route

The indolinone derivatives identified in our QSAR-based study as having favorable predicted pIC_50_ values (Table 4) were the subject of a proposed synthetic route, as shown in Scheme 2. This synthetic strategy was developed based on synthetic protocols previously reported in the scientific literature (Yu et al., 2019). Details regarding the chemicals, solvents, reaction conditions, and equipment used, as well as experimental procedures and spectral data, are available in the original publication (Yu et al., 2019).

The proposed synthetic route is hypothetical and is suggested based on related synthetic procedures reported in the literature. The proposed pathway is intended to illustrate a potential synthetic approach and does not represent an experimentally validated synthesis of the designed compounds. Therefore, experimental synthesis and structural characterization will be required to confirm the feasibility of the proposed route and the identity of the resulting compounds.

Pred 9 and Pred 10 were selected for further computational evaluation based on their favorable predicted pIC_50_ values in combination with their predicted physicochemical and pharmacokinetic properties. The selection was therefore not based solely on QSAR-predicted activity. However, because these compounds have not yet been synthesized or experimentally evaluated, their synthetic feasibility and biological activity remain to be experimentally confirmed.

### 3.7 Prediction of oral bioavailability and ADMET properties

The objective of this analysis was to evaluate the predicted oral bioavailability and ADMET properties of the newly designed compounds, Pred 9 and Pred 10, and to compare them with the most active compound from the original dataset. These *in silico* predictions were used to provide preliminary information regarding the physicochemical and pharmacokinetic properties of the compounds and to identify potential toxicity-related concerns. Figure 3 presents the predicted oral bioavailability profiles of Pred 9, Pred 10, and the reference compound M31.

**FIGURE 3:**
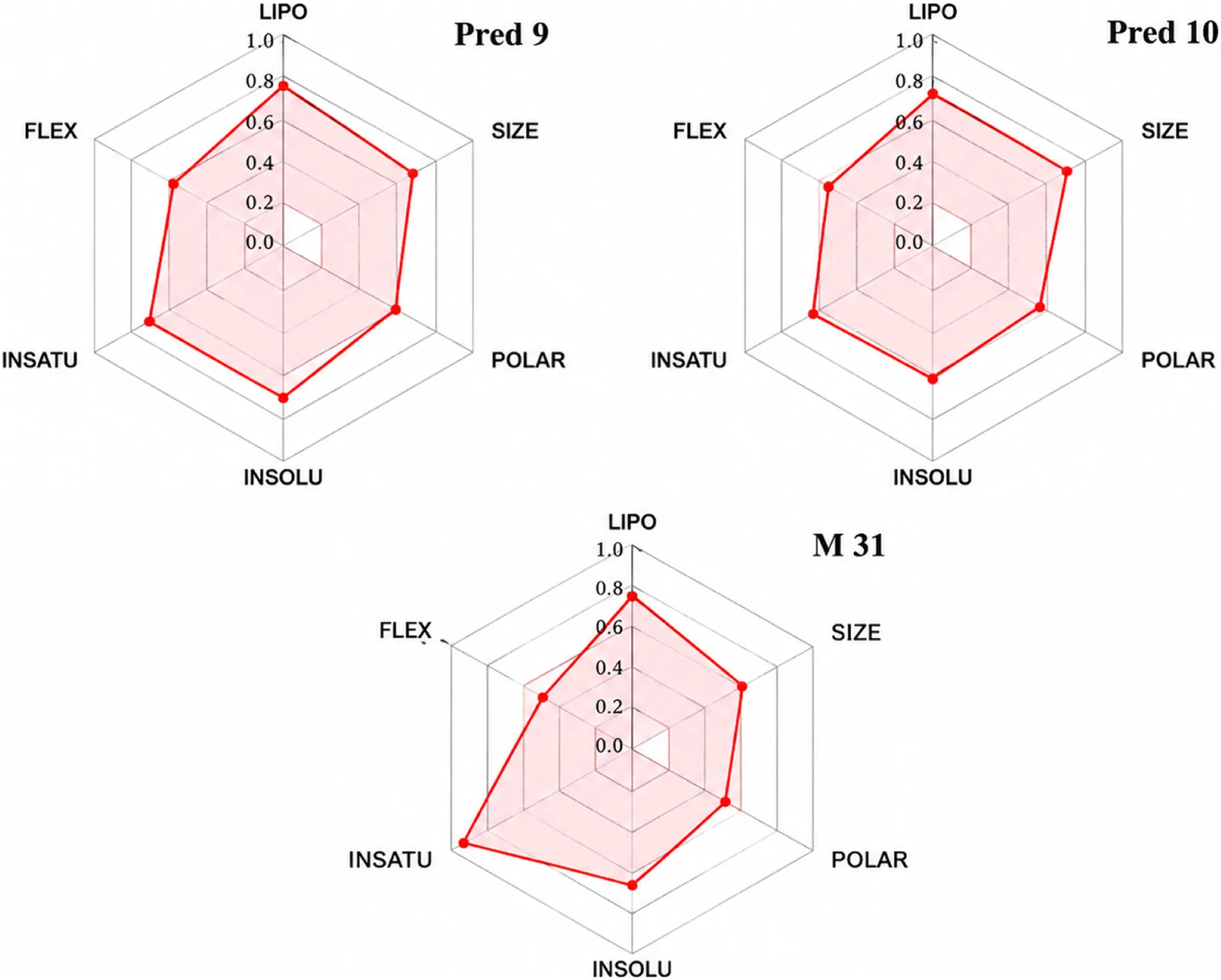
The oral bioavailability properties of the three molecules.

Figure 3 shows the profiles of six physicochemical and drug-likeness properties of Pred 9, Pred 10, and M31, namely LIPO (lipophilicity), SIZE (molecular size), POLAR (polarity), INSOLU (insolubility), INSATU (insaturation), and FLEX (flexibility). To obtain preliminary information on the drug-likeness, pharmacokinetic, and toxicity-related characteristics of the newly designed compounds, Pred 9 and Pred 10 were evaluated using *in silico* drug-likeness and ADMET prediction tools, together with M31, the most active compound in the original dataset, as a reference. The predicted ADMET properties of Pred 9, Pred 10, and M31 are summarized in Table 5. These computational predictions should be regarded as preliminary estimates and do not constitute experimental evidence; therefore, their actual pharmacokinetic and toxicity profiles require experimental validation.

**TABLE 5:** ADMET prediction results for Pred 9, Pred 10, and M31.

Molecule ID	Absorptions		Distributions			Metabolisms						Excretion	Toxicity
	Water solubility	Intestinal absorption	VDss	BBB	CNS	Substrate			Inhibitor			Total clearance	AMES toxicity
						CYP450							
						1A2	2D6	3A4	1A2	2D6	3A4		
	Numeric (log mol/L)	Numeric (%Absorbed)	Numeric (Log L/kg)	Numeric (Log BB)	Numeric (Log PS)	Categorical (+/−)						Numeric (Log ml/min/kg)	Categorical (Yes/No)
Pred 9	−3.472	92.44	−0.135	−0.75	−1.949	Yes	No	Yes	Yes	No	Yes	0.23	No
Pred 10	−3.139	82.57	0.103	−0.824	−2.295	Yes	No	Yes	Yes	No	Yes	0.239	No
M31	−4.688	88.529	−0.178	−0.109	−1.793	Yes	No	Yes	Yes	No	Yes	−0.067	Yes

### 3.8 Molecular docking studies

#### 3.8.1 Result of molecular docking

Molecular docking was employed to investigate the potential binding modes and ligand–receptor interactions of the proposed compounds within the MEK1 binding site (Er-rajy et al., 2023b; 2023a; 2025c; Zarougui et al., 2024; Lenda et al., 2025). The docking results provide structural insight into the possible interactions between the ligands and the target protein; however, docking scores alone cannot establish inhibitory potency or anticancer activity. Pred 9 and Pred 10 exhibited favorable predicted binding energies of −10.27 and −10.49 kcal mol^-1^, respectively. These results suggest potentially favorable interactions with the MEK1 binding site. The predicted binding poses and protein–ligand interactions were further examined. The molecular docking results for the two proposed molecules are presented in Figure 4.

**FIGURE 4:**
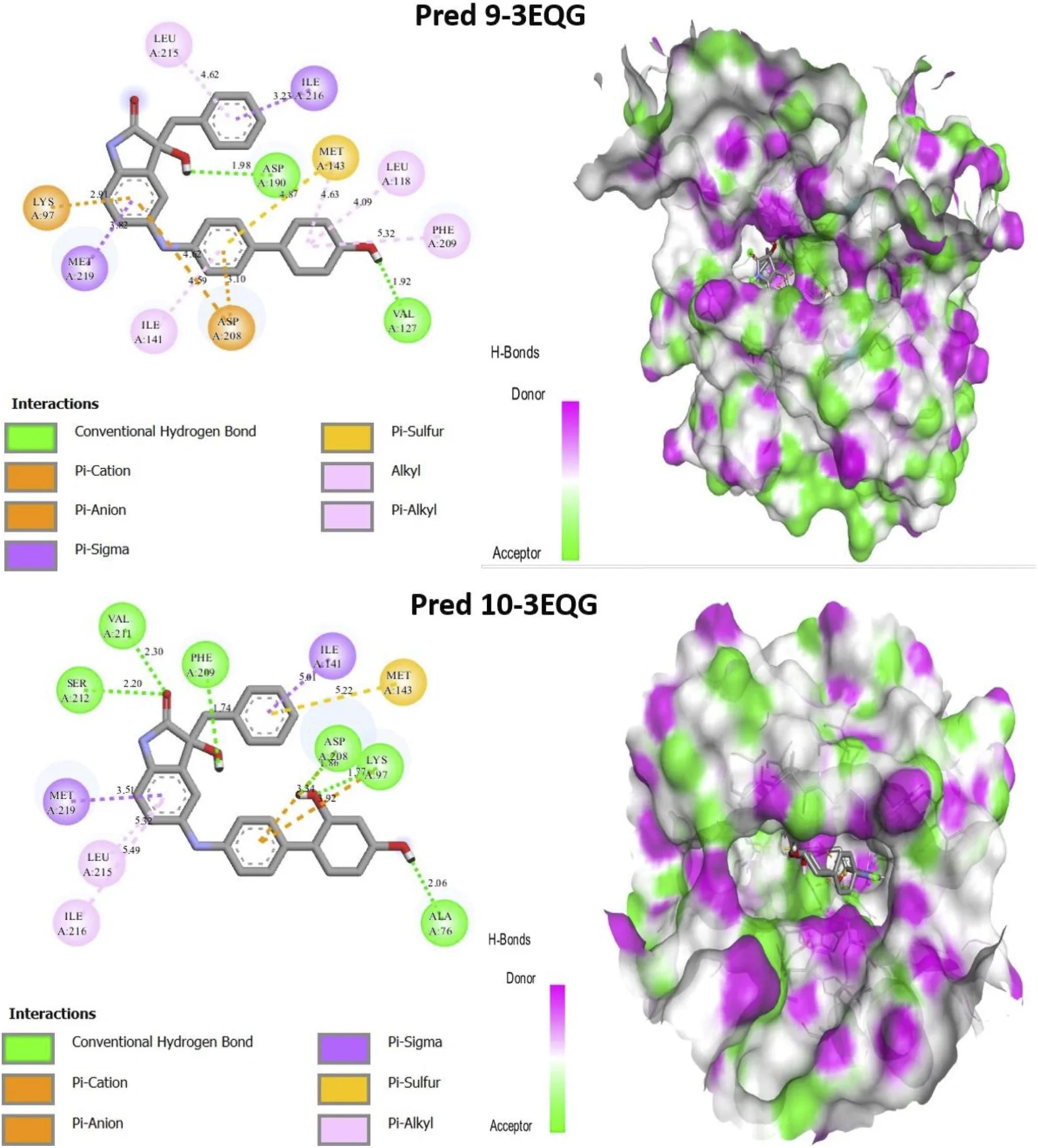
Two-dimensional (2D) and three-dimensional (3D) interactions of Pred 9 and Pred 10 with the 3EQG receptor.

#### 3.8.2 Results of the re-docking study

The reliability of the molecular docking protocol was evaluated through a re-docking procedure using the co-crystallized ligand of the MEK1 crystal structure (PDB ID: 3EQG). The native ligand was extracted from the crystallographic complex and re-docked into the original binding site using the same docking parameters applied to Pred 9 and Pred 10. The re-docked native ligand showed a predicted docking score of −8.4 kcal mol^-1^. As shown in Figure 5, the predicted binding pose was subsequently superimposed with the experimental crystallographic conformation. A root-mean-square deviation (RMSD) value of 0.90 Å was obtained between the experimental and re-docked ligand poses, indicating close agreement between the predicted and crystallographic binding conformations. This RMSD value, being below the commonly accepted threshold of 2.0 Å, supports the ability of the applied docking protocol to reproduce the experimentally observed binding mode.

**FIGURE 5:**
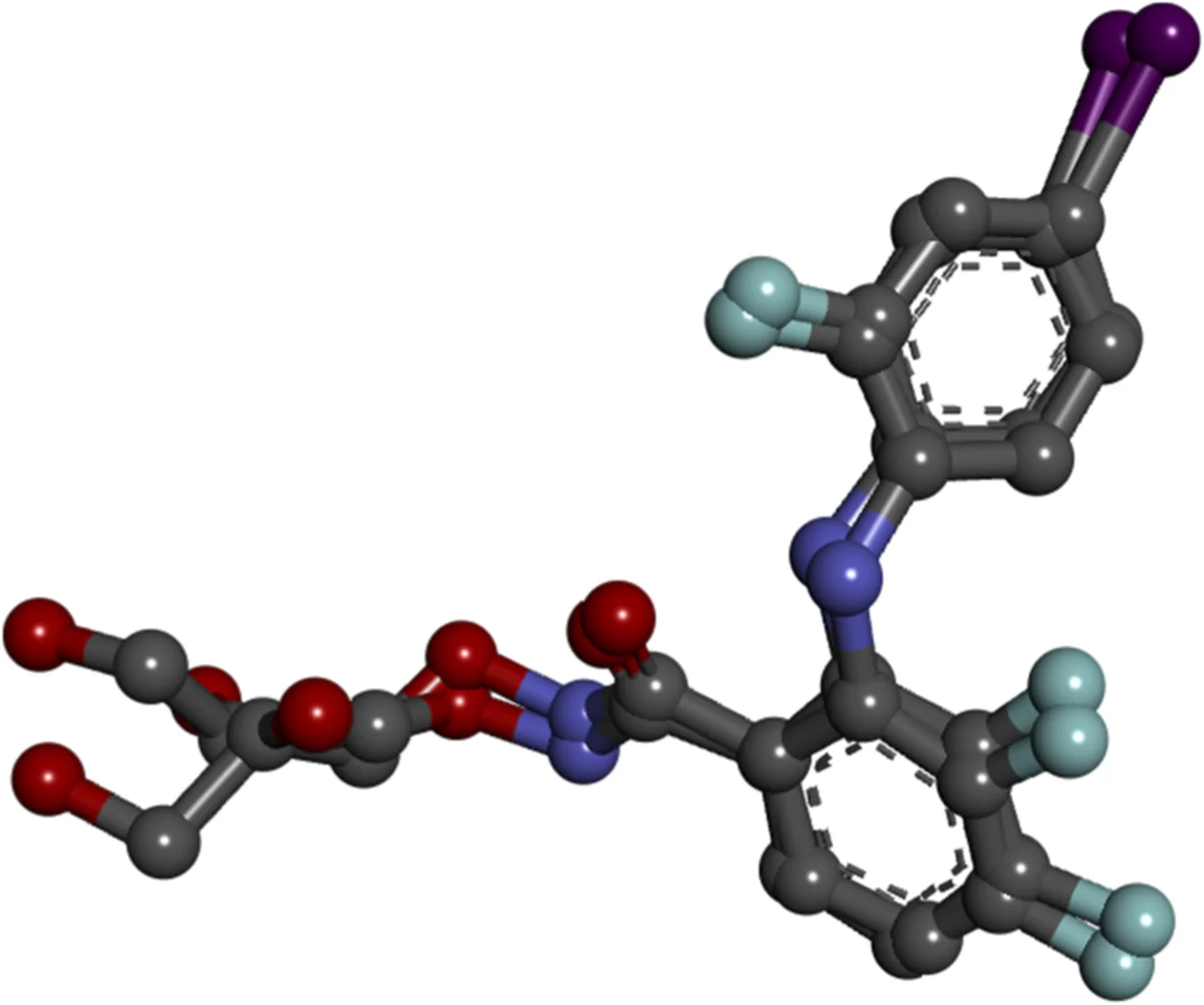
Superposition of the crystallographic pose of the co-crystallized ligand and the pose obtained after re-docking into the MEK1 binding.

### 3.9 MD results

MD simulations can be used to assess how stable a ligand remains in a protein active site and to identify which key amino acids contribute to the ligand–protein interaction. The conformational behavior of the Pred 9–3EQG and Pred 10–3EQG complexes was independently monitored over 100 ns using MD simulations. Protein-ligand interactions and the RMSD metric were used to evaluate the MD trajectories, as shown in Figure 6.

**FIGURE 6:**
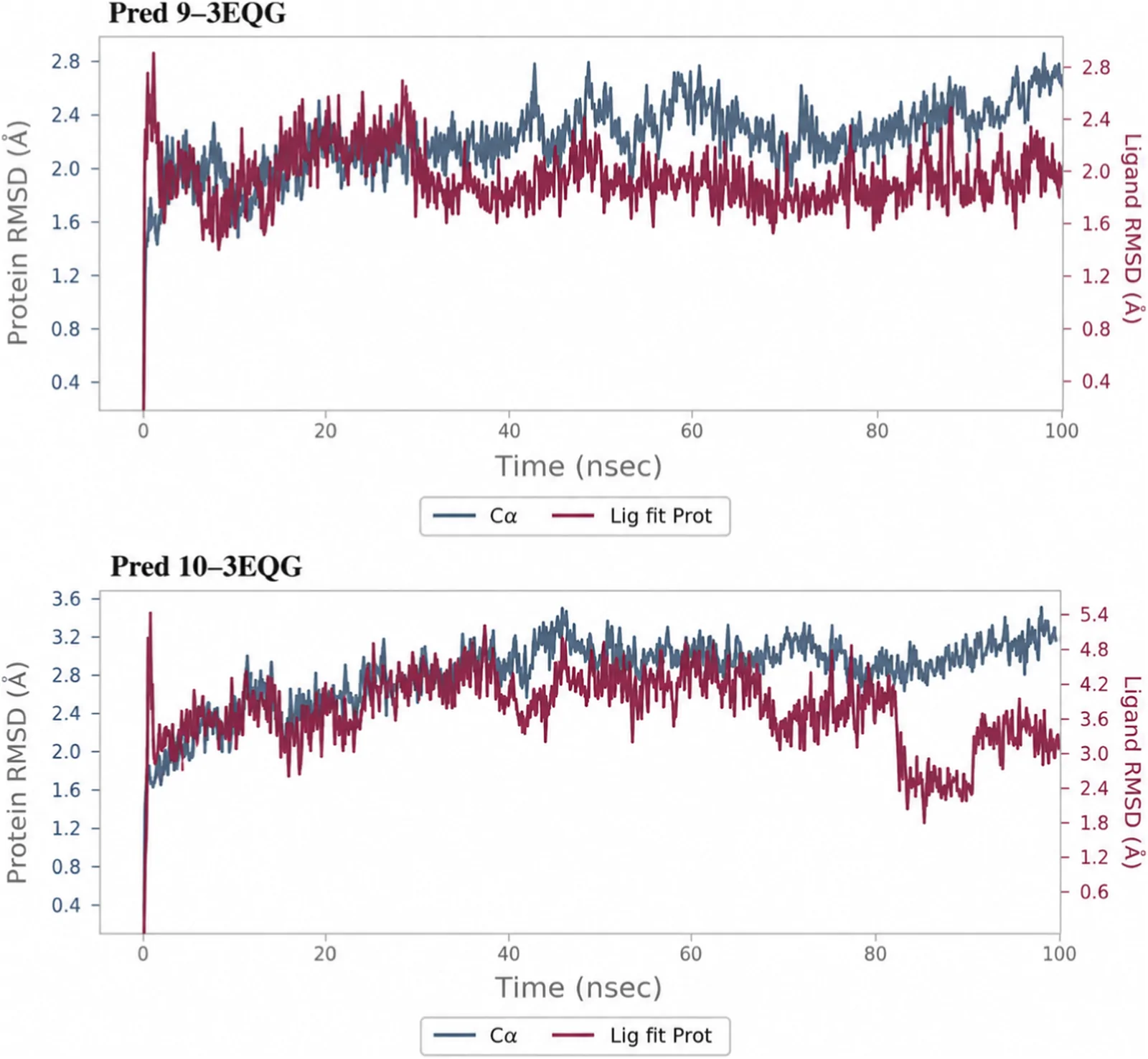
RMSD profiles of the Pred 9–3EQG and Pred 10–3EQG complexes during the 100 ns MD simulations.

Analysis of the interactions observed between the MEK1 structure (PDB ID 3EQG) and the two computationally prioritized compounds during a simulation revealed significant contacts (Figure 7).

**FIGURE 7:**
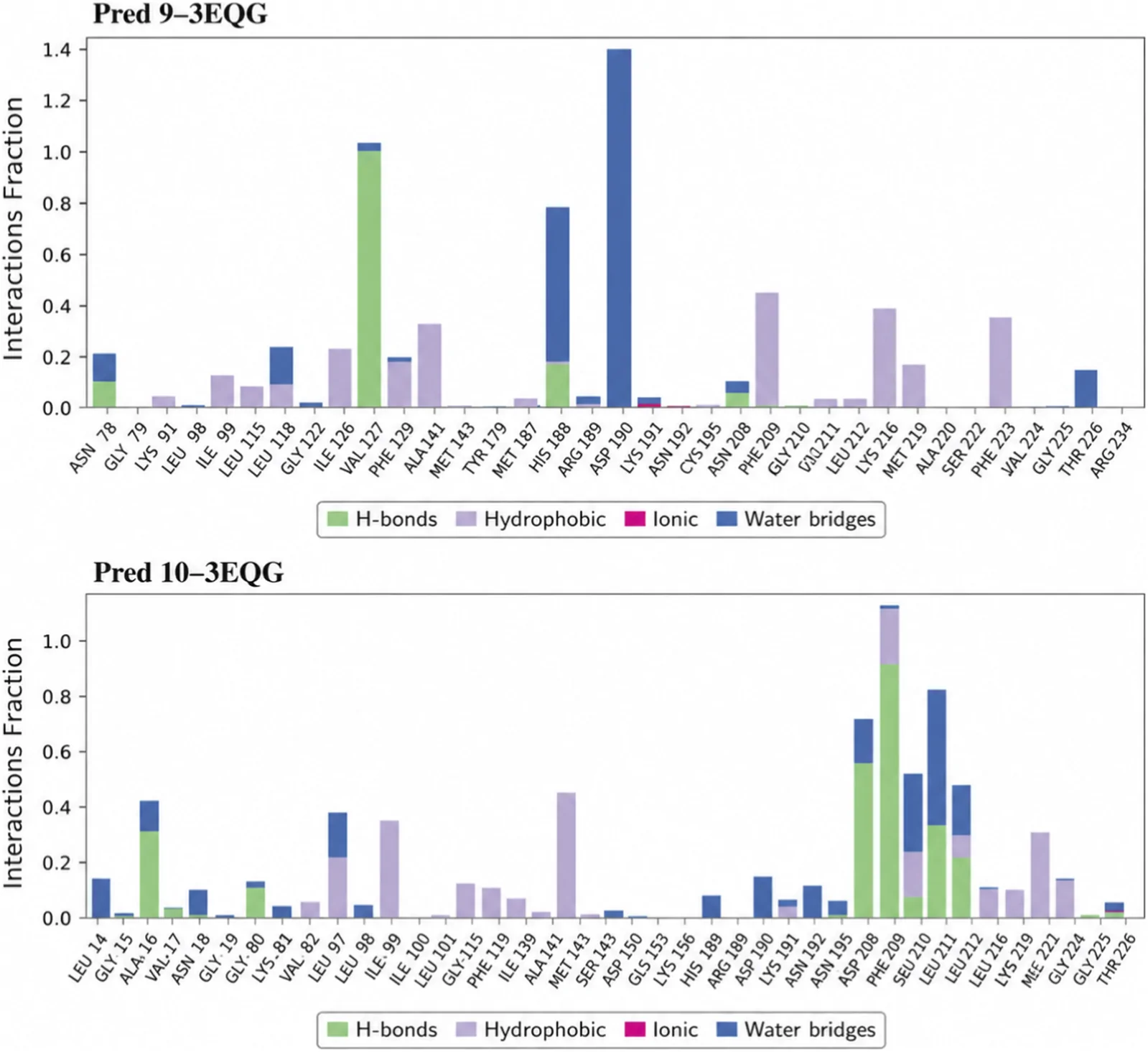
Protein–ligand contact profiles of the MEK1 structure (PDB ID 3EQG) in complex with Pred 9 and Pred 10 during the 100 ns MD simulations.

To further characterize the conformational behavior of the two protein–ligand complexes, additional MD analyses, including the radius of gyration (Rg), protein residue fluctuation (RMSF), and ligand atom fluctuations, were performed throughout the 100 ns simulations.

For the Pred 9–3EQG complex, the Rg profile shown in Figure 8 exhibited relatively limited fluctuations, with values mainly ranging from 5.3 to 5.6 Å, although occasional variations were observed during the simulation. The protein RMSF analysis indicated that most residues exhibited relatively low to moderate fluctuations, generally below approximately 2.5 Å. However, a limited number of residues displayed higher fluctuations, with a maximum RMSF value of approximately 5.4 Å, indicating localized regions of greater flexibility. Furthermore, the RMSF profile of the ligand atoms revealed moderate fluctuations ranging from approximately 0.8–2.3 Å, with most ligand atoms displaying RMSF values close to or below 1.5 Å.

**FIGURE 8:**
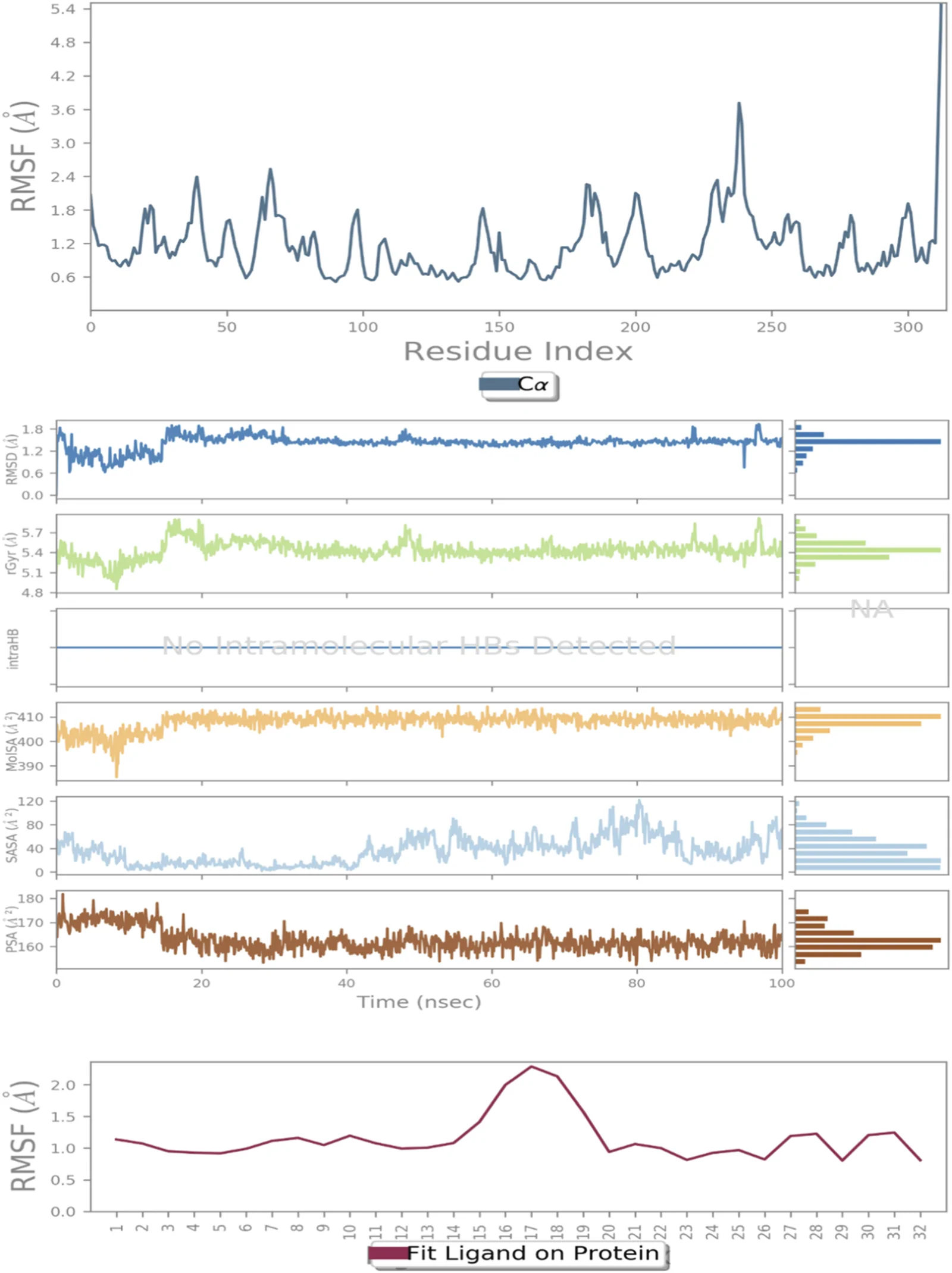
MD trajectory analysis of the Pred 9–3EQG complex, including RMSF, RMSD, radius of gyration (Rg), MolSA, SASA, PSA, and ligand RMSF profiles.

For the Pred 10–3EQG complex, the Rg profile shown in Figure 9 exhibited fluctuations throughout the 100 ns simulation, with values mainly ranging from 5.3 to 5.6 Å, although occasional variations were observed. The protein RMSF analysis indicated that most residues exhibited relatively limited fluctuations, generally below approximately 2.0 Å, whereas several localized regions showed higher flexibility, reaching a maximum of approximately 3.4 Å. In addition, the RMSF profile of the Pred 10 ligand atoms revealed fluctuations ranging from approximately 0.9–4.4 Å. Most ligand atoms exhibited moderate mobility, while higher fluctuations were observed for specific atoms, indicating differences in the flexibility of distinct ligand regions during the simulation.

**FIGURE 9:**
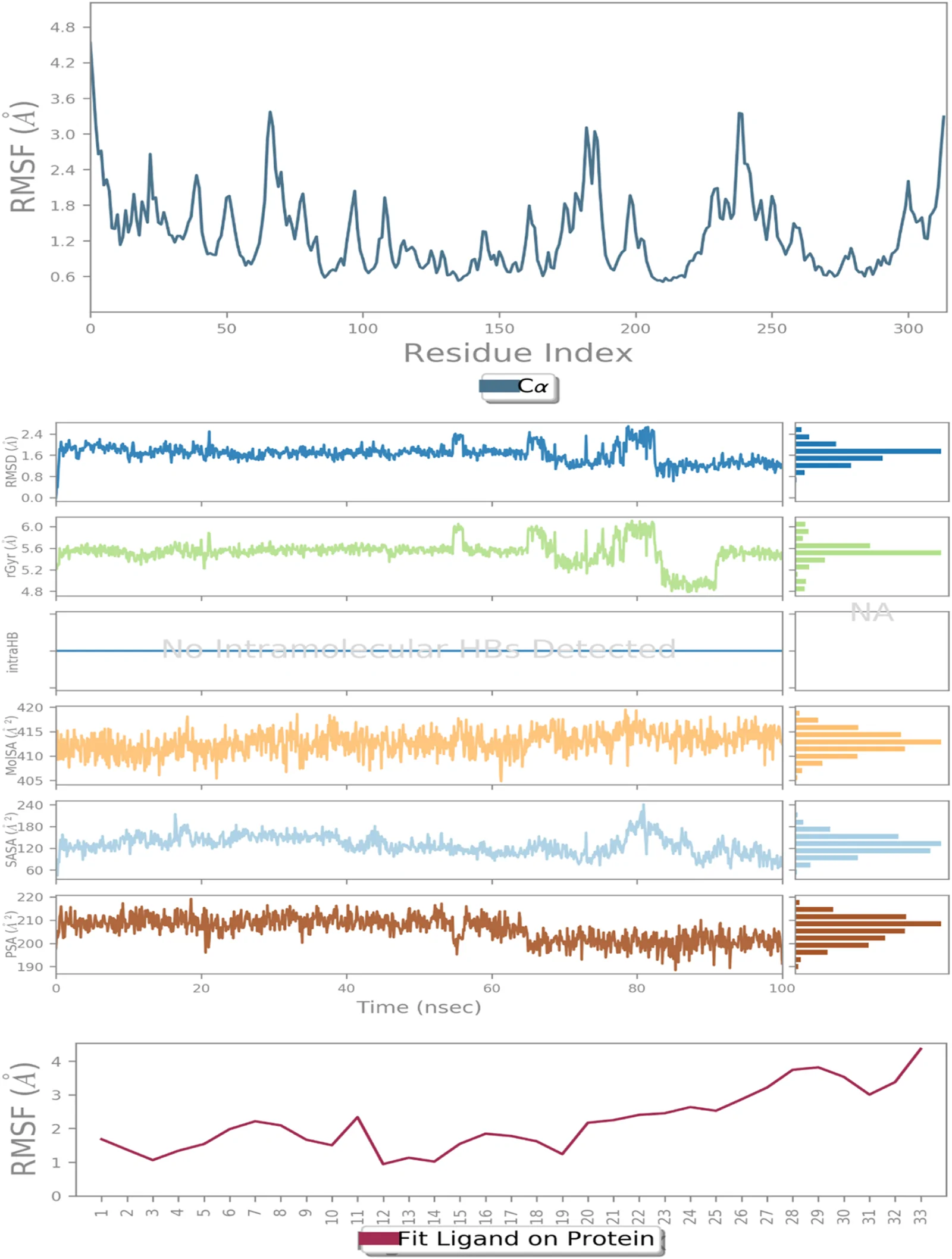
MD trajectory analysis of the Pred 10–3EQG complex, including RMSF, RMSD, radius of gyration (Rg), MolSA, SASA, PSA, and ligand RMSF profiles.

### 3.10 POM analysis

The POM theory (Petra, Osiris, and Molinspiration) provides a simplified approach for identifying and visualizing the pharmacophoric sites of the investigated compounds (Figure 10). It is particularly useful for gaining structural insights into molecular features that may contribute to their biological properties, rather than providing definitive predictions of biological activity. The POM analysis can therefore help identify pharmacophoric features potentially associated with antiviral, antifungal, antibacterial, or antitumor activities (Chalkha et al., 2022; Kawsar et al., 2022; Arzine et al., 2026c; 2026a; 2026b). As illustrated in Figure 11, the analysis revealed distinct pharmacophoric features in the investigated compounds; notably, Pred 9 and Pred 10 displayed features potentially associated with antitumor activity. These findings should be considered structure-based hypotheses that may help rationalize biological activity and guide the design of new analogues, rather than as definitive evidence of biological efficacy.

**FIGURE 10:**
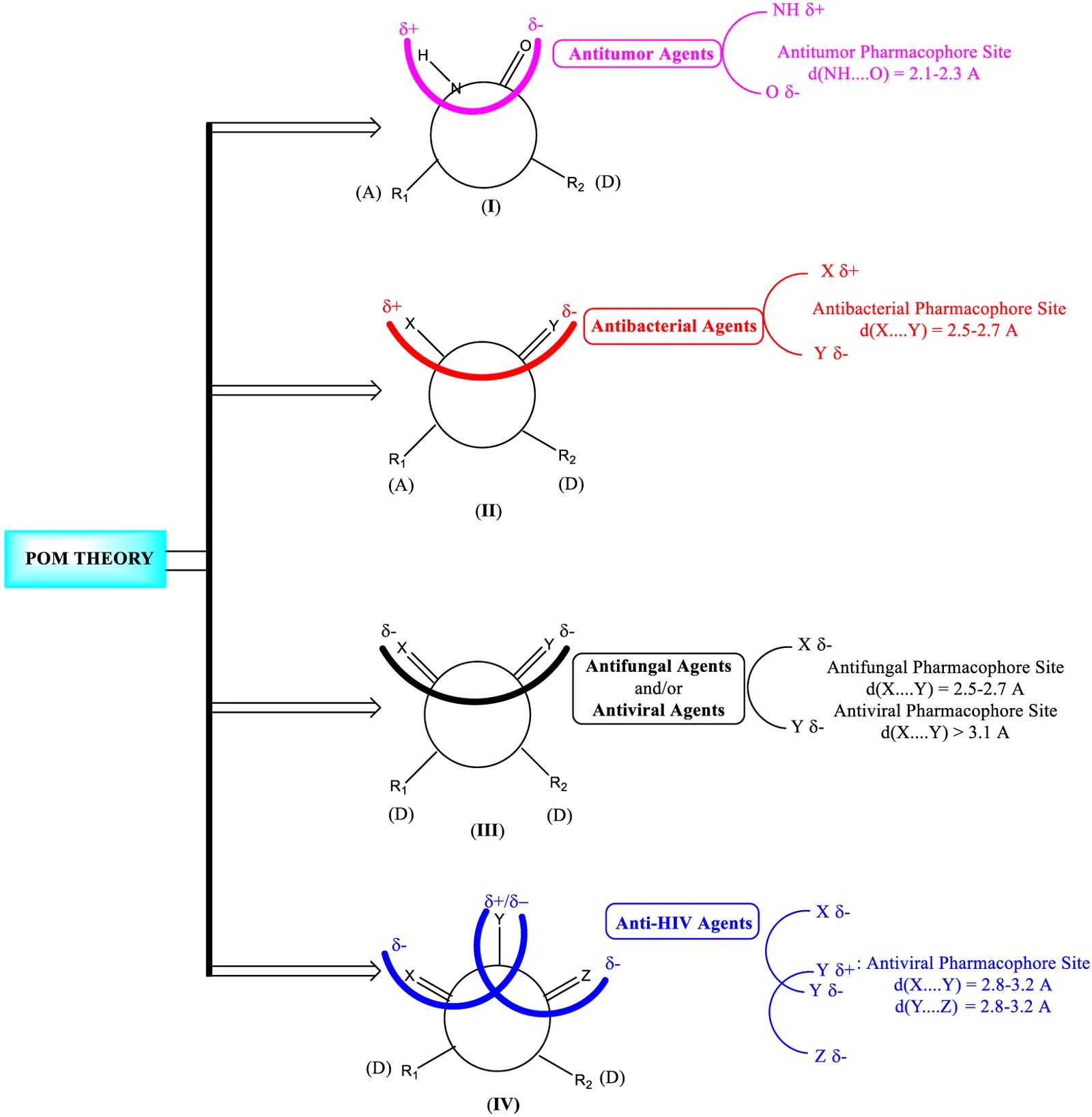
Conceptualization and enhancement of the locations of pharmacophores of diverse categories of medications.

**FIGURE 11:**
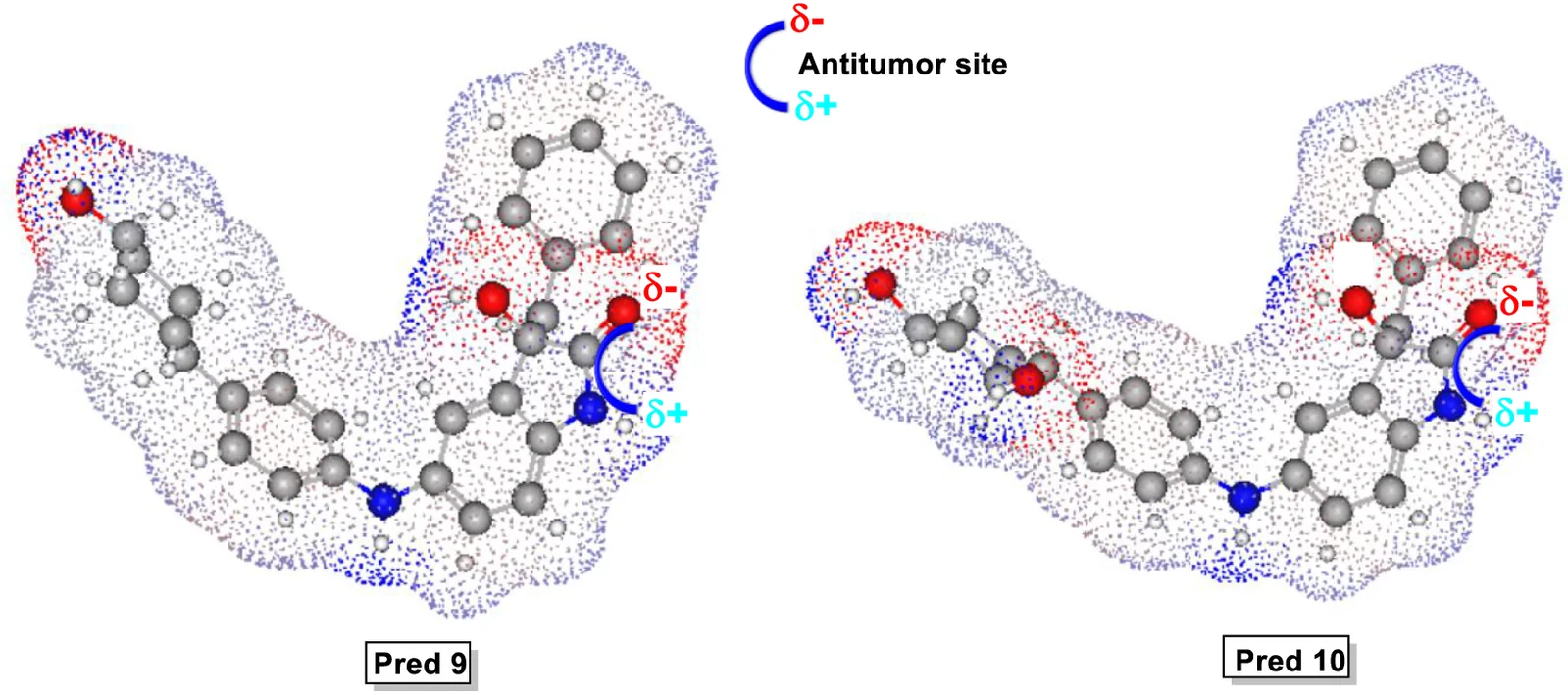
Identification of antitumor (O^δ–^, NH^δ+^) pharmacophore sites of *Pred* 9 and *Pred* 10 on the basis of POM Theory.

#### 3.10.1 Osiris calculation of toxicity and Drug-Score of Pred 9 and Pred 10

The toxicological properties and drug-likeness parameters of the two designed molecules, Pred 9 and Pred 10, were evaluated using the Osiris Property Explorer software. The results are presented in Table 6.

**TABLE 6:** Osiris calculations of the toxicity of *Pred* 9 and *Pred* 10.

Molecules ID	MUT	TUM	IRRI	REP	CLP	S	MW	TPSA	DL	D-S
Pred 9	---	+++	---	+++	4.69	−5.95	428.52	81.59	0.21	0.12
Pred 10	---	+++	---	+++	3.84	−5.56	444.52	101.8	0.5	0.15

Toxicity risks (+++): not toxic; (++): slightly toxic; (---): highly toxic. MUT: mutagenic; TUM: tumorigenic; IRRI: irritant; REP: reproductive effective. CLP: cLogP, S: solubility, TPSA: topological polar surface area, DL: Drug-likeness, DS: Drug-Score.

According to the Osiris predictions, both Pred 9 and Pred 10 showed no predicted carcinogenic (TUM) or reproductive toxicity (REP) risks. However, potential mutagenic (MUT) and irritant (IRRI) risks were indicated for both compounds. The predicted cLogP values were 4.69 for Pred 9 and 3.84 for Pred 10, while the Osiris predicted LogS values were −5.95 and −5.56, respectively.

Pred 9 and Pred 10 showed drug-likeness (DL) values of 0.21 and 0.50, respectively. The corresponding drug score (DS) values were 0.12 for Pred 9 and 0.15 for Pred 10.

#### 3.10.2 Molinspiration calculation of lipinski 5 rules and atomic charge of Pred 9 and Pred 10

The physicochemical properties, Lipinski’s Rule of Five parameters, atomic charge distributions, and molecular electrostatic potential (MEP) surfaces of Pred 9 and Pred 10 were calculated using Molinspiration. The results are presented in Table 7.

**TABLE 7:** Atomic charge and Molinspiration calculation of Lipinski’s Rule of Five of compounds *Pred* 9 and *Pred* 10.

Atomic charge and molinspiration calculation of Pred 9 and Pred 10		
Atomic charge	MEP surfaces	Lipinski 5 rules
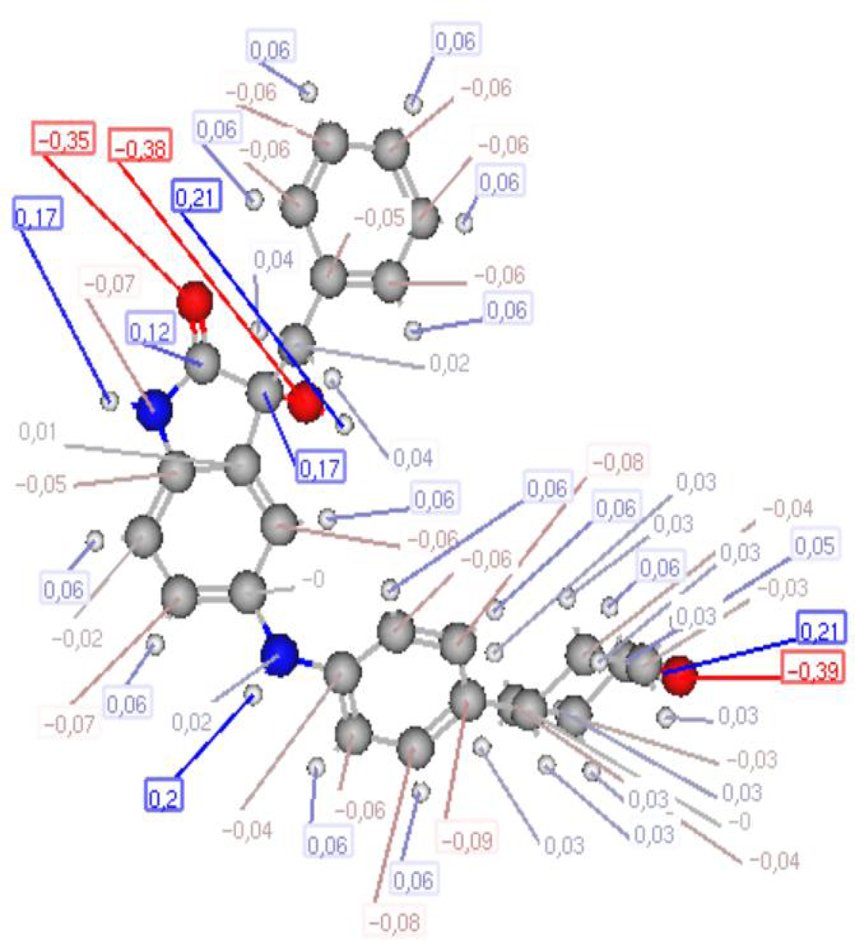 Pred 9	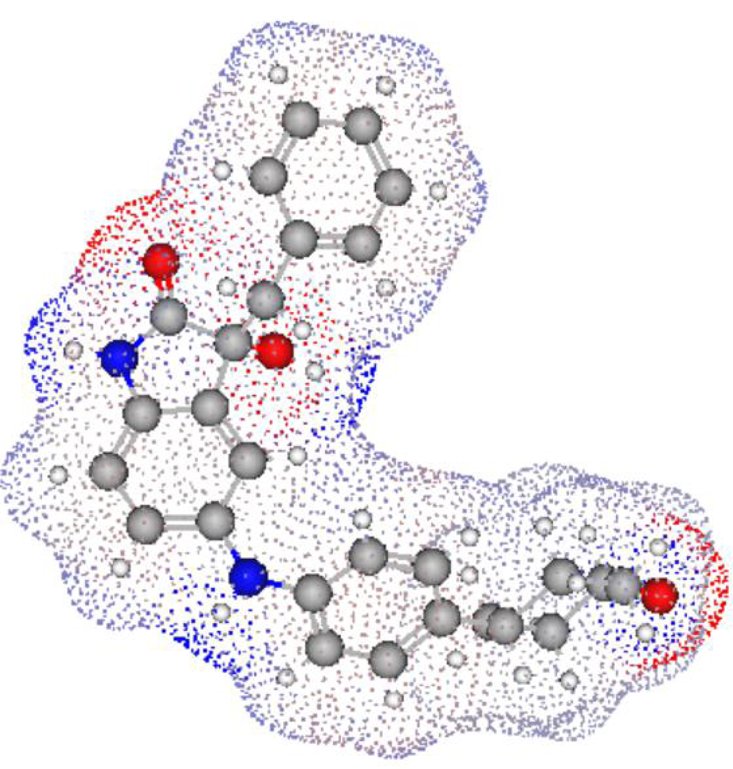	miLogP: 3.99 TPSA: 81.59 Å^2^ natoms: 32 MW: 428.52 g mol^-1^ nON: 3 nOHNH: 4 Nviolations: 0 nRotB: 5 Volume: 350 Å^3^
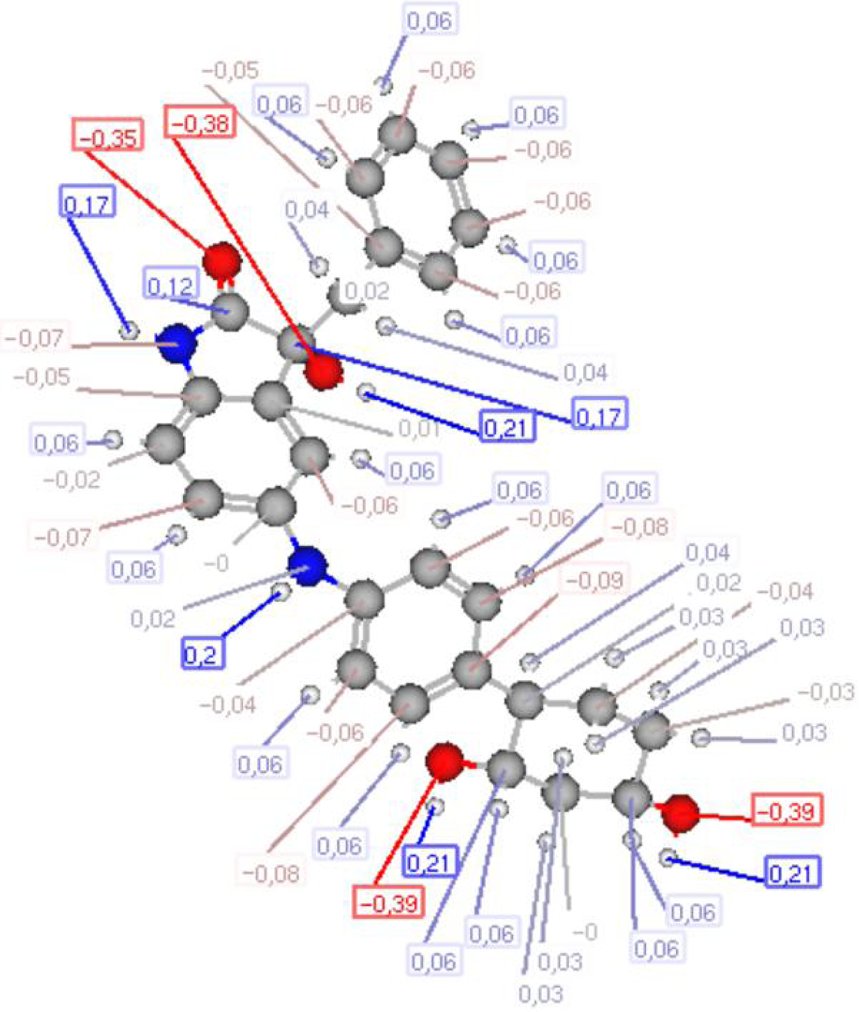 Pred 10	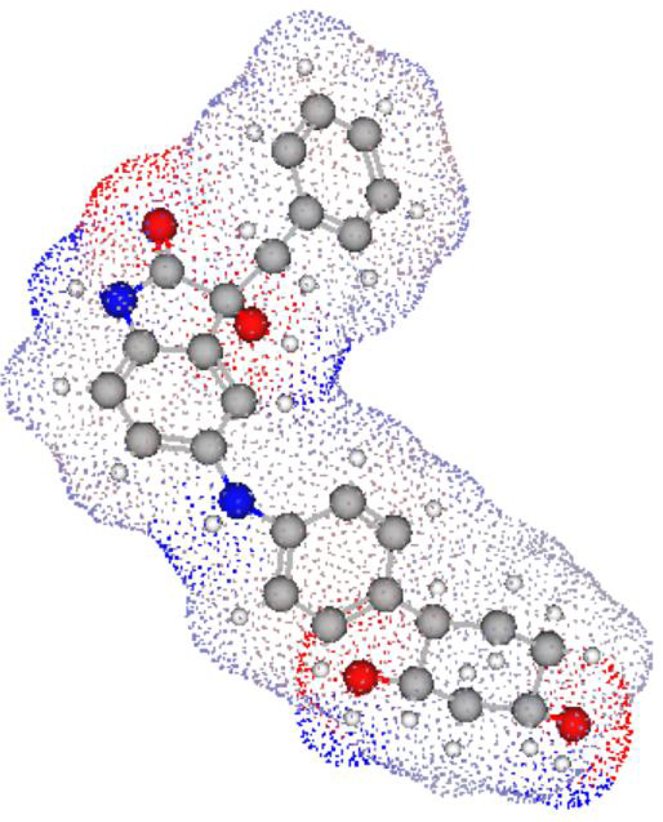	miLogP: 3.06 TPSA: 101.82 Å^2^ natoms: 33 MW: 444.52 gmol ^−1^ nON: 4 Nohnh: 5 nviolations: 0 nRotB: 5 Volume: 362 Å^3^

Table 7 shows the atomic charge distributions, MEP surfaces, and Lipinski’s Rule of Five parameters calculated using Molinspiration for molecules Pred 9 and Pred 10.

According to Table 7, the predicted miLogP values were 3.99 for Pred 9 and 3.06 for Pred 10. Their molecular weights were 428.52 and 444.52 g/mol, respectively. The TPSA values were 81.59 Å^2^ for Pred 9 and 101.82 Å^2^ for Pred 10. Also, Pred 9 and Pred 10 contained three and four hydrogen-bond acceptors (nON), respectively, whereas the numbers of hydrogen-bond donors (nOHNH) were four and five, respectively. Both compounds had five rotatable bonds (nRotB = 5). The calculated molecular volumes were approximately 350 Å^3^ for Pred 9 and 362 Å^3^ for Pred 10.

The atomic charge distribution identified negatively charged oxygen atoms associated with carbonyl groups and positively charged hydrogen atoms associated with NH groups in the indolinone scaffold. These regions were identified as potential pharmacophoric sites according to the POM analysis (Figure 11).

## 4 Discussion

### 4.1 MLR technique

From Equation 1, the MLR model relates the biological activity to the following descriptors: 1,4 VDW, Bend, Stretch-Bend, Total Valence Connectivity (TVC), Number of H-bond Acceptors (NHA), and Total Connectivity (TC), with a high R^2^ value and minimal error.

Next, by examining the coefficient normalization diagram (Figure 1), we observed that the developed model highlighted two of the most influential descriptors (TC and 1,4 VDW).

According to Table 1, we can state that the external validation criteria were satisfactorily met, suggesting that the developed QSAR model showed consistent performance across the applied validation methods. Therefore, the developed QSAR model demonstrated satisfactory statistical performance and showed potential for predicting the biological activity of structurally related compounds within the chemical space represented by the current dataset. However, considering the relatively limited dataset size, these predictions should be interpreted with caution and require further validation using additional experimental data.

The relationship between the experimental and predicted pIC_50_ values for the 33 compounds is illustrated in Figure 2. Overall, the predicted values showed reasonable agreement with the corresponding experimental activities, with most data points lying relatively close to the ideal line (y = x). The external test set, comprising molecules 1, 2, 6, 12, 13, and 23, also showed generally acceptable agreement between the experimental and predicted pIC_50_ values, supporting the model’s predictive performance for compounds not included in the model development process.

Nevertheless, several compounds exhibited noticeable deviations from the ideal line, indicating differences in prediction accuracy among individual molecules. These deviations may reflect the structural diversity of the dataset or molecular features that are not fully captured by the selected descriptors. Thus, although the graphical comparison provides a useful visual assessment of the model’s predictive behavior, it should not be considered independently of the statistical validation results. The numerical assessment of model performance is therefore complemented by the internal and external validation parameters and other diagnostic analyses described above.

### 4.2 Y-randomization test

The results of the Y-randomization test are presented in Table 2. The original QSAR model exhibited strong statistical performance, with an R^2^ value of 0.8739 and a Q^2^LOO value of 0.7736. In contrast, random permutation of the response variable resulted in a substantial decrease in model performance. The randomized models showed a mean R^2^ of 0.1871 and a mean Q^2^LOO of −0.3801. Although the maximum values obtained for the randomized models reached R^2^ = 0.6804 and Q^2^LOO = 0.5193, they remained lower than those of the original model. These results demonstrate that the statistical performance of the proposed QSAR model is unlikely to arise from chance correlations, thereby supporting its robustness and reliability.

### 4.3 Multicollinearity analysis

As shown in Table 3, the multicollinearity analysis provides additional support for the statistical robustness and interpretability of the developed QSAR model. The VIF values obtained for the six selected descriptors ranged from 1.98 to 5.12, indicating that severe linear dependence among the molecular descriptors was not observed. Five descriptors, namely Bend, Stretch-Bend, 1,4 VDW, NHA, and TVC, exhibited VIF values below 5, suggesting low to moderate multicollinearity (O’Brien, 2007). Although TC showed a slightly higher VIF value of 5.12, this value remained well below the commonly accepted threshold of 10 for severe multicollinearity. Therefore, the contribution of TC to the model is unlikely to result from excessive redundancy with the other descriptors. Overall, the results presented in Table 3 suggest that the selected descriptors provide complementary information related to different structural and physicochemical characteristics of the studied compounds. Consequently, the relationship between these descriptors and biological activity is less likely to be substantially affected by severe multicollinearity, supporting the robustness and interpretability of the developed QSAR model. Nevertheless, considering the relatively limited size of the dataset, these results should be interpreted within the chemical space represented by the studied compounds.

### 4.4 Design of new compounds

From the predicted activity results of the molecules presented in Table 4 predicted by the developed model, the results showed that the majority of them have a theoretical activity higher than that of the most active molecule in the initial series, as well as those proposed in our previous work (pIC_50_ = 5.0410). The two molecules that exhibit significant activity are the subject of further studies, such as molecular docking, MD simulations, and the prediction of ADMET properties, to identify their stability and similarity to existing drugs.

It is important to distinguish between the different types of evidence generated in the present study. The biological activity values of the original compounds included in the dataset were experimentally measured and were used for the development and validation of the QSAR model. In contrast, the pIC_50_ values obtained for Pred 9 and Pred 10 were generated using the developed QSAR equation and therefore represent predicted activities rather than experimentally determined values. Likewise, molecular docking provides predicted binding modes and potential protein–ligand interactions, MD simulations provide computational information regarding the conformational behavior of the simulated complexes, and the ADMET results represent predicted physicochemical, pharmacokinetic, and toxicity-related properties. These computational predictions require experimental validation.

### 4.5 Prediction of oral bioavailability and ADMET properties

In the oral bioavailability plots, the shaded area represents the optimal range for each evaluated physicochemical property (Er-Rajy et al., 2024; Hjouji et al., 2025). As shown in Figure 3, the proposed compounds, Pred 9 and Pred 10, were predicted to fall within the optimal range of the evaluated physicochemical properties. These *in silico* results suggest potentially favorable oral bioavailability characteristics compared with M 31; however, oral bioavailability has not been experimentally determined, and these predictions require experimental validation.

The predicted water-solubility values reported in Table 5 are expressed as logarithmic molar solubility (pkCSM predicted logS). On this scale, more negative pkCSM predicted logS values generally correspond to lower aqueous solubility. Therefore, the negative pkCSM predicted logS values should not be interpreted as indicating high or extreme water solubility. Pred 9 and Pred 10 showed pkCSM predicted logS values of −3.472 and −3.139, respectively, compared with −4.688 for M31, suggesting higher predicted aqueous solubility relative to M31. Nevertheless, the absolute aqueous solubility of these compounds remains to be experimentally determined, and limited solubility may still represent a potential limitation for formulation and oral bioavailability. Experimental solubility measurements are therefore required to validate these predictions. As shown in Table 5, the *in silico* model predicted high human intestinal absorption values exceeding 82% for the evaluated compounds. These results suggest a potential for favorable intestinal absorption; however, the actual absorption of these compounds remains to be experimentally determined.

Distribution can be described using the steady-state volume of distribution (VDss). A log VDss value below −0.15 suggests a relatively low volume of distribution, whereas values above 0 suggest a higher volume of distribution (Er-rajy et al., 2023f; Berrahou et al., 2026). A value of 0.45 indicates a relatively large distribution volume (Bouzammit et al., 2024; Arzine et al., 2026a). As reported in Table 5, the predicted VDss value for M 31 is below −0.15, suggesting a relatively low volume of distribution according to the applied classification. In comparison, Pred 9 and Pred 10 showed predicted VDss values between −0.15 and 0.45, suggesting a moderate volume of distribution. These predictions indicate potential differences in the distribution profiles of the compounds; however, the actual tissue distribution and clinical relevance of these differences require experimental investigation.

Blood-brain barrier (BBB) permeability is an important factor in the pharmaceutical industry because it affects a compound’s capacity to cross the BBB and potentially exert therapeutic effects within the central nervous system. According to the applied classification, predicted logBB values above 0.3 may indicate a relatively high capacity to cross the BBB, whereas values below −1 may suggest limited BBB penetration (Abraham, 2004; Er-rajy et al., 2023b). Based on the *in silico* predictions reported in Table 5, the examined compounds were predicted to exhibit an intermediate ability to cross the blood–brain barrier according to the applied classification thresholds. However, BBB permeability is influenced by multiple biological factors, and these computational predictions require experimental confirmation.

CNS permeability was estimated using the predicted logPS values. According to the applied classification, compounds with logPS values above −2 are generally considered more likely to penetrate the CNS, whereas values below −3 suggest limited CNS penetration (Ghose et al., 2012). Pred 9 and Pred 10 showed predicted logPS values of −1.949 and −2.295, respectively, suggesting differences in their predicted CNS permeability. Pred 9 may exhibit a relatively higher predicted ability to penetrate the CNS, whereas Pred 10 shows an intermediate predicted permeability between the commonly applied thresholds. However, these values represent computational predictions only and should not be interpreted as evidence of actual CNS penetration without experimental validation.

Cytochrome P450 enzymes play an important role in the metabolism of xenobiotics and may influence metabolic clearance and drug–drug interactions. According to the *in silico* predictions, Pred 9 and Pred 10 were predicted to act as substrates for CYP1A2 and CYP3A4, while they were predicted not to be substrates for CYP2D6 (Zanger and Schwab, 2013). In addition, both compounds were predicted to inhibit CYP1A2 and CYP3A4 but not CYP2D6. The predicted inhibition of CYP1A2 and CYP3A4 should be considered a potential limitation, as inhibition of these enzymes may alter the metabolism of co-administered drugs and increase the risk of drug–drug interactions. Therefore, these computational predictions require experimental confirmation using appropriate CYP inhibition and metabolic stability assays.

The predicted total clearance values were 0.230, 0.239, and −0.067 log mL/min/kg for Pred 9, Pred 10, and M31, respectively. These values suggest differences in the predicted elimination profiles among the compounds, with M31 showing the lowest predicted clearance value. However, total clearance and elimination kinetics cannot be reliably established from computational predictions alone. Experimental pharmacokinetic studies are required to determine the actual clearance and half-life of the compounds.

The *in silico* Ames toxicity model predicted M31 to be Ames-positive, whereas Pred 9 and Pred 10 were predicted to be Ames-negative. These results suggest that the designed compounds may have a lower predicted mutagenicity risk under the applied model compared with M31. However, an Ames-negative prediction should not be interpreted as evidence of overall safety or absence of toxicity, as the Ames test addresses only one aspect of potential toxicological risk. Comprehensive *in vitro* and *in vivo* toxicological studies are therefore required to establish the safety profiles of Pred 9 and Pred 10.

Overall, the ADMET analysis provides preliminary *in silico* information regarding the predicted physicochemical, pharmacokinetic, and toxicity-related properties of Pred 9 and Pred 10. Although some predicted characteristics, including relatively higher predicted aqueous solubility than that of M31, may support their prioritization for further investigation, their absolute solubility and overall pharmacokinetic properties remain to be experimentally determined. Potentially unfavorable characteristics should also be considered, particularly the predicted inhibition of CYP1A2 and CYP3A4, which may indicate a risk of altered drug metabolism and drug–drug interactions. Similarly, the higher predicted pIC_50_ values of Pred 9 and Pred 10 suggest potential improved activity according to the developed QSAR model but do not constitute evidence of superior biological activity or MEK1 inhibitory potency. Therefore, all computational predictions should be experimentally validated before conclusions regarding their oral bioavailability, pharmacokinetic performance, biological activity, toxicity, or overall safety can be established.

### 4.6 Molecular docking and re-docking studies

#### 4.6.1 Molecular docking

According to the results of the molecular docking shown in Figure 4, Pred 9 forms two hydrogen bonds with residues Val-127 and Asp-190 respectively, with distances of 1.92 Å and 1.98 Å, respectively. The residues Lys-97 and Asp-208 form two Pi-cation and Pi-anion bonds at distances of 2.91 Å and 3.10 Å, respectively. Pred 9 forms two Pi-sigma bonds with Ile-216 and Met-219 at 3.25 Å and 2.82 Å, respectively. There is a Pi-sulfur bond with the residue Met-143 at a distance of 4.63 Å. Finally, the molecule makes four alkyl and Pi-alkyl bonds with Phe-209, Leu-118, Ile-141, and Leu-125.

For Pred 10, there are six hydrogen bonds with the residues Val-211, Ser-212, Phe-209, Asp-208, Lys-97, and Ala-76, with distances of 2.30, 2.20, 1.74, 3.34, 1.77, and 2.06 Å, respectively. Additionally, there are two Pi-sigma bonds with the residues Ile-141 and Met-219, with distances of 5.01 Å and 3.51 Å, respectively. Furthermore, there is a Pi-sulfur bond with the residue Met-143 at a distance of 5.22 Å. Lastly, the molecule forms two Pi-alkyl bonds with the residues Leu-215 and Ile-216.

Previous studies have shown that MEK1 inhibitors and indolinone-based derivatives represent important scaffolds in anticancer drug discovery and have been investigated using both experimental and computational approaches (Ferreira et al., 2015; Nishal et al., 2023). In this context, the present study contributes to the computational exploration of structurally related compounds by combining QSAR modeling with molecular design, docking, MD simulations, and predicted pharmacokinetic properties. However, direct comparisons between the proposed compounds and previously reported MEK1 inhibitors should be made cautiously because differences in molecular structures, biological assays, experimental conditions, and computational protocols may influence the reported results. Consequently, the present findings should not be interpreted as demonstrating that Pred 9 and Pred 10 are superior to previously characterized MEK1 inhibitors without direct experimental comparison.

#### 4.6.2 Re-docking validation

As shown in Figure 5, the re-docking procedure resulted in an RMSD value of 0.90 Å between the experimental and re-docked ligand poses. This low RMSD value indicates close agreement between the crystallographic and re-docked conformations, suggesting that the applied docking protocol was able to reproduce the experimentally observed binding orientation of the native ligand. Since the obtained RMSD value is below the commonly used of 2.0 Å criterion for docking protocol validation, the results support the suitability of the selected docking parameters and binding-site definition (Er-rajy et al., 2022a; Er-rajy et al., 2023b). Therefore, the validated docking protocol was subsequently employed to investigate the potential binding modes and interactions of Pred 9 and Pred 10 within the binding site of the MEK1 protein structure (PDB ID 3EQG).

Overall, the molecular docking results suggest that Pred 9 and Pred 10 may interact favorably with residues located within the binding site of the target protein. These predicted interactions provide computational information regarding their potential binding modes and may support their prioritization for further investigation. However, molecular docking results alone cannot establish experimental binding affinity, inhibitory potency, or anticancer activity. Therefore, further experimental studies are required to confirm the actual biological activity and inhibitory potential of Pred 9 and Pred 10.

### 4.7 Molecular dynamics

For the complex Pred 9–3EQG, as shown in Figure 6, the RMSD value of the protein backbone showed a change in RMSD from 1.60 to 2.80 Å throughout the simulation. Throughout the 100 ns simulation, the RMSD of Pred 9 fluctuated from 1.60 to 2.80 Å, with relatively stable behavior being observed after approximately 30 ns (Figure 6). The average RMSD of the crystal structure complex was around 2.0 Å, while that of the Pred 9 was approximately 1.85 Å, suggesting relatively limited structural deviations during the simulation.

For the second complex Pred 10–3EQG, the RMSD of the protein backbone increased from 1.60 to 3.5 Å during the simulation, as seen in Figure 6. The protein backbone RMSD reached a relatively stable value of around 2.8 Å after approximately 30 ns and remained relatively stable throughout the remainder of the 100 ns simulation. Throughout the 100 ns simulation, the RMSD of Pred 10 fluctuated from approximately 1.60–3.50 Å (Figure 6). The average RMSD between the crystal structure complex and the designed molecule was approximately 2.5 Å, indicating relatively stable conformational behavior during the final stage of the simulation.

As illustrated in Figure 7, the protein–ligand contact analysis of the 3EQG complexes with the two computationally prioritized compounds, Pred 9 and Pred 10, revealed multiple interactions with amino acid residues throughout the 100 ns MD simulations. These interactions included hydrogen bonds, water-mediated bridges, and hydrophobic contacts, providing insights into the persistence of the predicted ligand–protein interactions throughout the simulations.

For the Pred 9–3EQG complex, Ile-126 exhibited a hydrogen bond for approximately 90% of the simulation time and a water-mediated bridge for about 10%. Asp-190 maintained a water-bridge interaction throughout the simulation (100%), whereas His-188 formed a hydrogen bond for approximately 90% of the simulation time and a water-mediated bridge interaction for approximately 20%. The persistence of these interactions suggests that hydrogen bonds and water-mediated contacts may contribute to maintaining the predicted association between Pred 9 and the protein binding region.

In the Pred 10–3EQG complex, Asp-208 formed a hydrogen bond for approximately 70% of the simulation time, while water-mediated interactions were observed for about 30%. Similarly, Phe-209 exhibited a hydrogen bond for approximately 70% of the simulation time and a hydrophobic interaction for approximately 20%. Ser-212 formed a hydrogen bond for approximately 40% of the simulation time and a water bridge for approximately 60%. These observations suggest that a combination of direct hydrogen bonds, water-mediated interactions, and hydrophobic contacts may contribute to the persistence of the predicted Pred 10–3EQG complex during the simulation.

Overall, both complexes exhibited sustained interactions with several amino acid residues throughout the simulation period. The differences in the interaction patterns between Pred 9 and Pred 10 may reflect differences in their predicted binding modes and conformational behavior within the protein binding region. When considered together with the RMSD and other molecular dynamics analyses, these interaction profiles provide complementary computational information regarding the stability of the predicted protein–ligand complexes. Nevertheless, these findings should be interpreted as computational predictions, and experimental studies are required to confirm the binding modes and biological activity of Pred 9 and Pred 10.

To provide a more comprehensive characterization of the simulated systems, additional analyses, including the radius of gyration (Rg), protein RMSF, and ligand RMSF, were performed for both complexes. The Rg profiles of the Pred 9–3EQG and Pred 10–3EQG complexes remained mainly within a comparable range, suggesting relatively limited variations in the overall compactness of the simulated systems, although occasional fluctuations were observed (Figures 8, 9).

The protein RMSF profiles indicated that most residues exhibited relatively low to moderate flexibility in both complexes. However, localized regions with higher RMSF values were observed, reflecting greater conformational flexibility in specific parts of the protein. The Pred 9–3EQG complex showed a higher maximum RMSF value than the Pred 10–3EQG complex, whereas most residues in both systems remained within relatively moderate fluctuation ranges (Figures 8, 9).

The ligand RMSF profiles also revealed differences in the conformational flexibility of the two designed compounds. Pred 9 exhibited relatively moderate ligand atom fluctuations, whereas Pred 10 showed higher fluctuations for specific atoms. These observations suggest that different regions of the two ligands may display different degrees of flexibility within the predicted MEK1 binding region during the simulation (Figures 8, 9).

Taken together with the RMSD profiles and the persistence of hydrogen-bond, water-mediated, and hydrophobic interactions, the Rg, protein RMSF, and ligand RMSF analyses provide complementary computational information regarding the conformational behavior and dynamic interactions of Pred 9 and Pred 10 within the MEK1 binding region. However, these computational results should be interpreted cautiously and do not by themselves demonstrate experimental binding affinity, MEK1 inhibition, or anticancer activity.

### 4.8 POM analysis

#### 4.8.1 Osiris calculation of toxicity and Drug-Score

The toxicological risk analysis showed that compounds Pred 9 and Pred 10 (Table 6) were not predicted to present TUM or REP risks according to the applied *in silico* model; however, potential IRRI and MUT risks were indicated. These predictions should be interpreted cautiously because different *in silico* toxicity models may provide different results depending on their underlying algorithms and toxicological endpoints. In particular, although Pred 9 and Pred 10 were predicted to be Ames-negative, the Osiris analysis indicated potential mutagenic and irritant risks. Therefore, based on the current computational predictions, the compounds cannot be considered definitively non-toxic or completely safe, and further experimental toxicological studies are required to establish their actual safety profiles. The predicted cLogP values of 4.69 for Pred 9 and 3.84 for Pred 10 indicate relatively high lipophilicity, which may favor membrane partitioning and permeability. The lower cLogP value of Pred 10 may suggest a comparatively more favorable predicted balance between lipophilicity and aqueous solubility. The predicted aqueous solubility was low, with Osiris-predicted LogS values of −5.95 for Pred 9 and −5.56 for Pred 10. The low predicted water solubility may be related, at least in part, to the structural characteristics of these compounds, including their aromatic features. Pred 10 therefore showed slightly higher predicted aqueous solubility than Pred 9. However, these values represent computational predictions and require experimental confirmation. With regard to the parameters considered by the Osiris model, Pred 10 exhibited a higher DL value of 0.50 than that of Pred 9 (0.21). Similarly, its DS was slightly higher (0.15 vs. 0.12). These results suggest that Pred 10 may exhibit a comparatively more favorable predicted balance of the physicochemical and toxicity-related parameters considered by the model. However, these computational scores should not be interpreted as evidence of superior pharmacological efficacy or therapeutic potential.

#### 4.8.2 Molinspiration calculation of lipinski 5 rules and atomic charge

The POM analysis identified negatively charged oxygen atoms associated with carbonyl groups and positively charged hydrogen atoms associated with NH groups as potential regions of pharmacophoric interest (Figure 11). These charge distributions may influence intermolecular interactions and could be relevant to the predicted structure–activity relationships of the indolinone derivatives. However, their direct contribution to MEK1 inhibition or anticancer activity cannot be established from the present computational analysis and requires experimental validation.

Several limitations of the present study should be acknowledged. First, the QSAR model was developed using a relatively limited dataset of 33 compounds, with 27 compounds used for model development and 6 compounds used for external validation. Although satisfactory internal and external validation results, Y-randomization analysis, and multicollinearity assessment support the statistical reliability of the model, the limited dataset size and the small external test set may restrict its generalizability. Therefore, the predicted activities of Pred 9 and Pred 10 should be interpreted cautiously and within the chemical and biological space represented by the original dataset. Second, molecular docking predicts possible ligand binding modes and protein–ligand interactions but does not directly measure binding affinity, enzyme inhibition, or cellular anticancer activity. Similarly, molecular dynamics simulations provide information regarding the conformational behavior of the simulated complexes under the selected computational conditions but cannot independently establish biological efficacy or therapeutic potential. Therefore, these methods should be regarded as complementary computational approaches rather than definitive evidence of MEK1 inhibition.

Finally, the ADMET and physicochemical properties reported in this study are computational predictions. In particular, the predicted water-solubility values, expressed as pkCSM predicted logS, provide estimates of aqueous solubility based on computational models and should not be considered experimentally measured solubility values. Although Pred 9 and Pred 10 showed higher predicted aqueous solubility than M31, their absolute solubility remains unknown and requires experimental determination. Experimental enzymatic, cellular, pharmacokinetic, solubility, and toxicity studies will therefore be necessary to validate the predicted properties and biological potential of Pred 9 and Pred 10.

## 5 Future directions

Single-agent treatments that target the RAF–MEK–ERK signaling pathway are either already used in clinical practice or being tested in clinical trials, including inhibitors of BRAF and MEK. Because signaling pathways can overlap and downstream targets can be activated through alternative routes, these single-agent treatments often provide clinical benefit for only about 6–8 months before acquired drug resistance develops (McCubrey et al., 2007; Degirmenci et al., 2021).

The activation of the RAF-MEK-ERK pathway and the relative expression of its components, may differ across patients and may also change within the same patient depending on disease stage or the local tumor microenvironment. Because cancer cells can change and tumor signaling is complex, treatment often needs more than one approach, such as combining therapies or trying alternative options, to reduce resistance and improve patient outcomes (Lim et al., 2017; Ullah et al., 2022). The results obtained so far for the two molecules remain preliminary and should be considered provisional at this stage (Pred 9 and Pred 10). Further work is planned, including chemical synthesis and *in vivo* testing of their activity, to confirm whether they have therapeutic efficacy against cancer.

## 6 Conclusion

This study highlights the relevance of the RAF-MEK-ERK signaling pathway in cancer development and provides a computational investigation specifically focused on MEK1 as a molecular target. Our computational approach, which integrated 2D-QSAR modeling, molecular docking, molecular dynamics simulations, POM analysis, and *in silico* ADMET prediction, was used to investigate the structural and physicochemical features associated with the reported biological activity of the studied indolinone derivatives. The QSAR model was evaluated using complementary internal and external validation approaches and subsequently used to guide the design of ten new computational candidates. Among them, Pred 9 and Pred 10 showed higher predicted activity according to the developed QSAR model and were therefore computationally prioritized for further investigation. In silico ADMET analysis suggested that these compounds may possess favorable predicted pharmacokinetic properties. A potential synthetic route for the designed compounds was also proposed based on previously reported procedures. Molecular docking, molecular dynamics simulations, and POM analysis provided complementary computational information regarding the predicted interactions of Pred 9 and Pred 10 with the MEK1 binding region and the conformational behavior of the resulting predicted ligand–protein complexes. However, these findings should be interpreted cautiously, as Pred 9 and Pred 10 are computationally designed candidates that have not yet been synthesized or experimentally evaluated. The present study has several important limitations. The QSAR model was developed using a relatively limited dataset, which may restrict the generalizability of its predictions. Furthermore, the newly designed compounds were evaluated entirely using computational approaches, and no experimental data are currently available to confirm their MEK1 inhibitory activity, anticancer activity, pharmacokinetic behavior, toxicity, or overall safety. Therefore, the present findings should be regarded as predictive and hypothesis-generating rather than as evidence of therapeutic efficacy. Overall, this study provides a computational framework for exploring the structure–activity relationships of indolinone derivatives and prioritizing candidate molecules for further experimental investigation.

## Data Availability

The original contributions presented in the study are included in the article/Supplementary Material, further inquiries can be directed to the corresponding authors.
