# Supplementary material for: In silico design of novel indolinone-based anticancer agents and proposal of synthetic routes
Source: Front Bioinform. 2026 Oct 14;6:1964610. doi: 10.3389/fbinf.2026.1964610 (PMC13713695; doi:10.3389/fbinf.2026.1964610)
Supplement: Supplementary file 1 [file Table1.docx]

***Supplementary Material***

1 **Supplementary Data**

**Table S1:** Data to be analyzed using the QSAR method.

| **Molecules ID** | **R1** | **R2** | **IC_50_** | **pIC_50_** |
| --- | --- | --- | --- | --- |
| 1* | p-Me |  | 19.00 | 4.7212 |
| 2* | p-Me |  | 45.50 | 4.3420 |
| 3 | p-Cl |  | 50.90 | 4.2933 |
| 4 | p-Cl |  | 13.20 | 4.8794 |
| 5 | p-Cl |  | 13.50 | 4.8697 |
| 6* | p-Cl |  | 24.60 | 4.6091 |
| 7 | p-Me |  | 55.30 | 4.2573 |
| 8 | p-Me |  | 63.60 | 4.1965 |
| 9 | p-Cl |  | 48.70 | 4.3125 |
| 10 | p-Cl |  | 36.70 | 4.4353 |
| 11 | p-Cl |  | 16.20 | 4.7905 |
| 12* | p-Me |  | 31.80 | 4.4976 |
| 13* | p-Me |  | 22.70 | 4.6440 |
| 14 | p-Me |  | 31.60 | 4.5003 |
| 15 | p-Me |  | 14.70 | 4.8327 |
| 16 | p-F |  | 32.50 | 4.4881 |
| 17 | p-F |  | 16.80 | 4.7747 |
| 18 | p-F |  | 50.70 | 4.2950 |
| 19 | p-F |  | 18.10 | 4.7423 |
| 20 | p-F |  | 80.40 | 4.0947 |
| 21 | p-Cl |  | 24.40 | 4.6126 |
| 22 | p-Cl |  | 40.80 | 4.3893 |
| 23* | p-Cl |  | 25.00 | 4.6021 |
| 24 | p-Cl |  | 30.30 | 4.5186 |
| 25 | p-Cl |  | 20.20 | 4.6946 |
| 26 | p-Cl |  | 26.60 | 4.5751 |
| 27 | p-CF_3_ |  | 12.00 | 4.9208 |
| 28 | p-CF_3_ |  | 16.30 | 4.7878 |
| 29 | p-CF_3_ |  | 17.20 | 4.7645 |
| 30 | p-CF_3_ |  | 18.80 | 4.7258 |
| 31 | p-CF_3_ |  | 9.10 | 5.0410 |
| 32 | p-CF_3_ |  | 18.20 | 4.7399 |
| 33 | o-Cl |  | 14.60 | 4.8356 |

*Test set.

**Table S2:** The descriptor sizes calculated for the 33 compounds.

| **Molecule ID** | **IC_50_** | **pIC_50_** | **Bend** | **Stretch-Bend** | **1,4 VDW ^a^** | **N H A ^b^** | **T C ^c^** | **T V C ^d^** |
| --- | --- | --- | --- | --- | --- | --- | --- | --- |
| 1* | 19.00 | 4.7212 | 21.08 | 0.09 | 14.31 | 2.00 | 3.34E-04 | 4.92E-06 |
| 2* | 45.50 | 4.3420 | 21.91 | 0.16 | 17.90 | 2.00 | 6.82E-05 | 1.00E-06 |
| 3 | 50.90 | 4.2933 | 21.44 | 0.13 | 14.79 | 2.00 | 2.73E-04 | 4.56E-06 |
| 4 | 13.20 | 4.8794 | 21.82 | 0.17 | 17.70 | 2.00 | 6.82E-05 | 1.14E-06 |
| 5 | 13.50 | 4.8697 | 23.50 | 0.11 | 19.57 | 2.00 | 6.82E-05 | 3.58E-07 |
| 6* | 24.60 | 4.6091 | 29.06 | 0.02 | 19.86 | 4.00 | 1.61E-05 | 3.16E-08 |
| 7 | 55.30 | 4.2573 | 16.69 | 0.06 | 14.59 | 2.00 | 2.73E-04 | 5.68E-06 |
| 8 | 63.60 | 4.1965 | 21.51 | -0.13 | 18.43 | 4.00 | 1.61E-05 | 3.95E-08 |
| 9 | 48.70 | 4.3125 | 16.61 | 0.07 | 14.38 | 2.00 | 2.73E-04 | 6.44E-06 |
| 10 | 36.70 | 4.4353 | 21.46 | -0.12 | 18.24 | 4.00 | 1.61E-05 | 4.48E-08 |
| 11 | 16.20 | 4.7905 | 17.63 | 0.09 | 16.22 | 4.00 | 1.45E-04 | 7.78E-07 |
| 12* | 31.80 | 4.4976 | 16.80 | 0.10 | 13.05 | 3.00 | 2.89E-04 | 2.70E-06 |
| 13* | 22.70 | 4.6440 | 17.18 | 0.15 | 13.62 | 3.00 | 2.36E-04 | 2.20E-06 |
| 14 | 31.60 | 4.5003 | 16.51 | 0.04 | 17.11 | 3.00 | 5.91E-05 | 1.73E-07 |
| 15 | 14.70 | 4.8327 | 22.33 | -0.07 | 17.53 | 5.00 | 1.39E-05 | 1.53E-08 |
| 16 | 32.50 | 4.4881 | 16.66 | 0.10 | 12.29 | 4.00 | 2.89E-04 | 1.02E-06 |
| 17 | 16.80 | 4.7747 | 17.04 | 0.14 | 12.87 | 4.00 | 2.36E-04 | 8.32E-07 |
| 18 | 50.70 | 4.2950 | 16.84 | 0.14 | 13.66 | 4.00 | 1.45E-04 | 5.09E-07 |
| 19 | 18.10 | 4.7423 | 16.36 | 0.04 | 16.36 | 4.00 | 5.91E-05 | 6.54E-08 |
| 20 | 80.40 | 4.0947 | 22.20 | -0.07 | 16.76 | 6.00 | 1.39E-05 | 5.78E-09 |
| 21 | 24.40 | 4.6126 | 16.73 | 0.11 | 12.85 | 3.00 | 2.89E-04 | 3.06E-06 |
| 22 | 40.80 | 4.3893 | 16.81 | 0.13 | 13.54 | 3.00 | 2.05E-04 | 2.16E-06 |
| 23* | 25.00 | 4.6021 | 17.10 | 0.15 | 13.43 | 3.00 | 2.36E-04 | 2.50E-06 |
| 24 | 30.30 | 4.5186 | 16.90 | 0.15 | 14.21 | 3.00 | 1.45E-04 | 1.53E-06 |
| 25 | 20.20 | 4.6946 | 16.42 | 0.05 | 16.92 | 3.00 | 5.91E-05 | 1.96E-07 |
| 26 | 26.60 | 4.5751 | 22.26 | -0.06 | 17.32 | 5.00 | 1.39E-05 | 1.73E-08 |
| 27 | 12.00 | 4.9208 | 17.15 | 0.12 | 13.13 | 6.00 | 1.45E-04 | 7.28E-08 |
| 28 | 16.30 | 4.7878 | 17.24 | 0.15 | 13.81 | 6.00 | 1.02E-04 | 5.15E-08 |
| 29 | 17.20 | 4.7645 | 17.52 | 0.17 | 13.72 | 6.00 | 1.18E-04 | 5.94E-08 |
| 30 | 18.80 | 4.7258 | 17.33 | 0.17 | 14.50 | 6.00 | 7.23E-05 | 3.64E-08 |
| 31 | 9.10 | 5.0410 | 16.86 | 0.06 | 17.20 | 6.00 | 2.95E-05 | 4.67E-09 |
| 32 | 18.20 | 4.7399 | 22.69 | -0.05 | 17.61 | 8.00 | 6.96E-06 | 4.13E-10 |
| 33 | 14.60 | 4.8356 | 19.33 | 0.11 | 17.31 | 3.00 | 5.91E-05 | 1.96E-07 |

*****Test set, **^a^ 1,4 VdW energy, ^b^** Number of H-Bond donor, **^c^** Total connectivity, **^d^** Total valence connectivity

**Table S3** Complete results of the 500 Y-randomization (Y-scrambling) runs performed for the QSAR model, including the corresponding R² and Q² values obtained after random permutation of the dependent variable.

| Random | R2 | Q2_LOO | Random | R2 | Q2_LOO | Random | R2 | Q2_LOO | Random | R2 | Q2_LOO | Random | R2 | Q2_LOO |
| --- | --- | --- | --- | --- | --- | --- | --- | --- | --- | --- | --- | --- | --- | --- |
| 1 | 0,047799 | -0,77451 | 101 | 0,066255 | -0,48158 | 201 | 0,213699 | -0,4842 | 301 | 0,275621 | -0,33383 | 401 | 0,126436 | -0,50805 |
| 2 | 0,210478 | -0,29602 | 102 | 0,411453 | 0,053101 | 202 | 0,244845 | -0,28226 | 302 | 0,313005 | -0,22247 | 402 | 0,253494 | -0,22941 |
| 3 | 0,139348 | -0,43318 | 103 | 0,4317 | -0,07722 | 203 | 0,091218 | -0,57201 | 303 | 0,040667 | -0,74485 | 403 | 0,122608 | -0,50877 |
| 4 | 0,27707 | -0,1472 | 104 | 0,198442 | -0,36858 | 204 | 0,197495 | -0,23512 | 304 | 0,067366 | -0,76296 | 404 | 0,118856 | -0,61247 |
| 5 | 0,398172 | -0,01294 | 105 | 0,104298 | -0,70799 | 205 | 0,536578 | 0,219093 | 305 | 0,269215 | -0,10025 | 405 | 0,078559 | -0,70193 |
| 6 | 0,113253 | -0,66281 | 106 | 0,259706 | -0,1832 | 206 | 0,037117 | -0,89945 | 306 | 0,222147 | -0,2304 | 406 | 0,056663 | -0,57426 |
| 7 | 0,090531 | -0,66331 | 107 | 0,614322 | 0,427754 | 207 | 0,307051 | -0,31523 | 307 | 0,104888 | -0,61391 | 407 | 0,200717 | -0,27578 |
| 8 | 0,375952 | -0,00429 | 108 | 0,049601 | -0,59515 | 208 | 0,234338 | -0,42297 | 308 | 0,308104 | -0,05026 | 408 | 0,282892 | -0,24966 |
| 9 | 0,121572 | -0,41534 | 109 | 0,259953 | -0,19207 | 209 | 0,246315 | -0,37271 | 309 | 0,083671 | -0,63427 | 409 | 0,185501 | -0,27439 |
| 10 | 0,20304 | -0,19787 | 110 | 0,266775 | -0,15207 | 210 | 0,172541 | -0,44214 | 310 | 0,085581 | -0,45536 | 410 | 0,328664 | -0,14647 |
| 11 | 0,098249 | -0,34867 | 111 | 0,385972 | -0,05884 | 211 | 0,391288 | -0,01629 | 311 | 0,081311 | -0,71065 | 411 | 0,109628 | -0,48246 |
| 12 | 0,254882 | -0,22515 | 112 | 0,178376 | -0,47532 | 212 | 0,026111 | -0,60771 | 312 | 0,091714 | -0,40282 | 412 | 0,341856 | -0,01656 |
| 13 | 0,207156 | -0,26095 | 113 | 0,211883 | -0,4196 | 213 | 0,149788 | -0,58236 | 313 | 0,212047 | -0,32798 | 413 | 0,062718 | -0,50941 |
| 14 | 0,522103 | 0,231957 | 114 | 0,190269 | -0,46194 | 214 | 0,078088 | -0,70517 | 314 | 0,20825 | -0,48114 | 414 | 0,183915 | -0,24981 |
| 15 | 0,295028 | -0,0903 | 115 | 0,624963 | 0,271172 | 215 | 0,263347 | -0,06527 | 315 | 0,233322 | -0,28607 | 415 | 0,217224 | -0,1887 |
| 16 | 0,293742 | -0,14274 | 116 | 0,1579 | -0,44858 | 216 | 0,122671 | -0,50758 | 316 | 0,160705 | -0,48684 | 416 | 0,113513 | -0,47451 |
| 17 | 0,249155 | -0,42523 | 117 | 0,140581 | -0,31895 | 217 | 0,232247 | -0,18577 | 317 | 0,252807 | -0,23071 | 417 | 0,088472 | -0,47284 |
| 18 | 0,153594 | -0,36137 | 118 | 0,091219 | -0,76504 | 218 | 0,224426 | -0,18488 | 318 | 0,103058 | -0,60876 | 418 | 0,048846 | -0,68407 |
| 19 | 0,14844 | -0,39719 | 119 | 0,334562 | -0,05426 | 219 | 0,178818 | -0,45281 | 319 | 0,318841 | -0,09324 | 419 | 0,168452 | -0,21818 |
| 20 | 0,146814 | -0,40705 | 120 | 0,048944 | -0,671 | 220 | 0,090627 | -0,59213 | 320 | 0,173989 | -0,49524 | 420 | 0,028267 | -0,70321 |
| 21 | 0,336944 | -0,16556 | 121 | 0,218221 | -0,45809 | 221 | 0,129987 | -0,60014 | 321 | 0,193322 | -0,3374 | 421 | 0,177701 | -0,25138 |
| 22 | 0,188087 | -0,2364 | 122 | 0,134608 | -0,63681 | 222 | 0,442781 | -0,07892 | 322 | 0,205811 | -0,41168 | 422 | 0,195886 | -0,36763 |
| 23 | 0,132432 | -0,53888 | 123 | 0,103622 | -0,33097 | 223 | 0,204558 | -0,37581 | 323 | 0,298447 | -0,12581 | 423 | 0,454741 | 0,03466 |
| 24 | 0,189991 | -0,26645 | 124 | 0,134661 | -0,47423 | 224 | 0,017191 | -0,60426 | 324 | 0,333694 | -0,02945 | 424 | 0,15493 | -0,65867 |
| 25 | 0,211074 | -0,33403 | 125 | 0,284965 | -0,20665 | 225 | 0,09415 | -0,72817 | 325 | 0,14344 | -0,50564 | 425 | 0,096556 | -0,67626 |
| 26 | 0,177484 | -0,34079 | 126 | 0,21467 | -0,33837 | 226 | 0,160104 | -0,40648 | 326 | 0,447355 | 0,071877 | 426 | 0,090566 | -0,6453 |
| 27 | 0,346559 | -0,03541 | 127 | 0,098319 | -0,45728 | 227 | 0,160811 | -0,30261 | 327 | 0,303388 | -0,04334 | 427 | 0,146787 | -0,34599 |
| 28 | 0,107716 | -0,44823 | 128 | 0,212384 | -0,35744 | 228 | 0,374629 | 0,024378 | 328 | 0,188793 | -0,43709 | 428 | 0,178555 | -0,35229 |
| 29 | 0,077874 | -0,57186 | 129 | 0,074513 | -0,36263 | 229 | 0,161886 | -0,44043 | 329 | 0,400437 | -0,29119 | 429 | 0,05292 | -0,8176 |
| 30 | 0,275954 | -0,23122 | 130 | 0,298524 | -0,15145 | 230 | 0,360426 | -0,11522 | 330 | 0,163243 | -0,30933 | 430 | 0,118047 | -0,44733 |
| 31 | 0,149902 | -0,40563 | 131 | 0,312236 | -0,13021 | 231 | 0,076226 | -0,86082 | 331 | 0,078027 | -0,56869 | 431 | 0,106389 | -0,52237 |
| 32 | 0,227026 | -0,32649 | 132 | 0,15973 | -0,20965 | 232 | 0,277646 | -0,21053 | 332 | 0,275815 | -0,08645 | 432 | 0,310436 | -0,06229 |
| 33 | 0,255804 | -0,07108 | 133 | 0,118732 | -0,61182 | 233 | 0,279542 | -0,15552 | 333 | 0,212457 | -0,46433 | 433 | 0,142806 | -0,48688 |
| 34 | 0,049544 | -0,75145 | 134 | 0,499167 | 0,223172 | 234 | 0,068513 | -0,55881 | 334 | 0,154013 | -0,27062 | 434 | 0,288972 | -0,05948 |
| 35 | 0,114119 | -0,65172 | 135 | 0,148158 | -0,55121 | 235 | 0,078358 | -0,48894 | 335 | 0,321066 | -0,07538 | 435 | 0,114535 | -0,37929 |
| 36 | 0,235364 | -0,26914 | 136 | 0,10317 | -0,60199 | 236 | 0,158706 | -0,32751 | 336 | 0,05744 | -0,75977 | 436 | 0,211812 | -0,26002 |
| 37 | 0,21352 | -0,17676 | 137 | 0,124983 | -0,54088 | 237 | 0,100982 | -0,5305 | 337 | 0,119761 | -0,61619 | 437 | 0,376716 | 0,004838 |
| 38 | 0,217459 | -0,27593 | 138 | 0,139134 | -0,37736 | 238 | 0,151125 | -0,41819 | 338 | 0,193205 | -0,57385 | 438 | 0,107831 | -0,4494 |
| 39 | 0,113513 | -0,49593 | 139 | 0,084641 | -0,51866 | 239 | 0,052526 | -0,65535 | 339 | 0,093414 | -0,61825 | 439 | 0,041224 | -0,73871 |
| 40 | 0,274774 | -0,13841 | 140 | 0,134707 | -0,41339 | 240 | 0,241645 | -0,17436 | 340 | 0,257566 | -0,401 | 440 | 0,082028 | -0,51555 |
| 41 | 0,354131 | -0,00868 | 141 | 0,127009 | -0,39269 | 241 | 0,435316 | 0,150949 | 341 | 0,048878 | -0,47556 | 441 | 0,451136 | 0,070218 |
| 42 | 0,155252 | -0,31283 | 142 | 0,026181 | -0,57664 | 242 | 0,221078 | -0,20687 | 342 | 0,359791 | -0,3252 | 442 | 0,417121 | -0,02192 |
| 43 | 0,324655 | -0,12206 | 143 | 0,26937 | -0,0677 | 243 | 0,249955 | -0,43545 | 343 | 0,091371 | -0,79947 | 443 | 0,329538 | -0,03379 |
| 44 | 0,210644 | -0,40071 | 144 | 0,118668 | -0,50801 | 244 | 0,220454 | -0,25799 | 344 | 0,483671 | 0,07034 | 444 | 0,149942 | -0,26136 |
| 45 | 0,276329 | -0,1637 | 145 | 0,212524 | -0,43465 | 245 | 0,293287 | -0,25186 | 345 | 0,231155 | -0,34298 | 445 | 0,343902 | -0,0261 |
| 46 | 0,075552 | -0,58175 | 146 | 0,194864 | -0,34578 | 246 | 0,124565 | -0,56483 | 346 | 0,094172 | -0,54161 | 446 | 0,083578 | -0,71119 |
| 47 | 0,074832 | -0,42476 | 147 | 0,317264 | -0,16995 | 247 | 0,11547 | -0,55194 | 347 | 0,39364 | -1,4E-05 | 447 | 0,326776 | -0,19854 |
| 48 | 0,173758 | -0,53266 | 148 | 0,176529 | -0,33104 | 248 | 0,365069 | -0,3118 | 348 | 0,097294 | -0,34072 | 448 | 0,325139 | -0,12191 |
| 49 | 0,379746 | 0,075789 | 149 | 0,217688 | -0,28308 | 249 | 0,289427 | -0,26504 | 349 | 0,164825 | -0,41976 | 449 | 0,093482 | -0,52439 |
| 50 | 0,117981 | -0,3828 | 150 | 0,267827 | -0,11374 | 250 | 0,146151 | -0,40899 | 350 | 0,167341 | -0,39369 | 450 | 0,12318 | -0,66099 |
| 51 | 0,205539 | -0,35644 | 151 | 0,093097 | -0,4207 | 251 | 0,260299 | -0,24325 | 351 | 0,076095 | -0,58202 | 451 | 0,245197 | -0,25329 |
| 52 | 0,299311 | -0,19204 | 152 | 0,431077 | 0,064776 | 252 | 0,263025 | -0,21867 | 352 | 0,262504 | -0,18194 | 452 | 0,208517 | -0,34361 |
| 53 | 0,202225 | -0,30493 | 153 | 0,262312 | -0,22281 | 253 | 0,154852 | -0,38312 | 353 | 0,437646 | 0,159794 | 453 | 0,129953 | -0,54474 |
| 54 | 0,12569 | -0,4551 | 154 | 0,293334 | -0,25614 | 254 | 0,361965 | 0,016138 | 354 | 0,323465 | -0,15877 | 454 | 0,258166 | -0,36147 |
| 55 | 0,209478 | -0,39657 | 155 | 0,15198 | -0,49194 | 255 | 0,207574 | -0,26482 | 355 | 0,216186 | -0,14281 | 455 | 0,194339 | -0,46135 |
| 56 | 0,297119 | -0,1615 | 156 | 0,184081 | -0,32213 | 256 | 0,177956 | -0,22691 | 356 | 0,215289 | -0,23977 | 456 | 0,140007 | -0,54846 |
| 57 | 0,191175 | -0,45564 | 157 | 0,258522 | -0,22072 | 257 | 0,033603 | -0,48487 | 357 | 0,167967 | -0,52762 | 457 | 0,321616 | -0,02936 |
| 58 | 0,313197 | -0,19438 | 158 | 0,105765 | -0,4999 | 258 | 0,099544 | -0,55418 | 358 | 0,12502 | -0,41051 | 458 | 0,129183 | -0,39024 |
| 59 | 0,164968 | -0,45837 | 159 | 0,13795 | -0,387 | 259 | 0,077652 | -0,51921 | 359 | 0,186753 | -0,50091 | 459 | 0,274574 | -0,35565 |
| 60 | 0,186714 | -0,49853 | 160 | 0,05807 | -0,76065 | 260 | 0,242768 | -0,29794 | 360 | 0,123217 | -0,57995 | 460 | 0,128521 | -0,58028 |
| 61 | 0,256289 | -0,32302 | 161 | 0,153445 | -0,26172 | 261 | 0,099867 | -0,38563 | 361 | 0,13417 | -0,5095 | 461 | 0,051546 | -0,62969 |
| 62 | 0,154556 | -0,31397 | 162 | 0,289186 | -0,11921 | 262 | 0,236386 | -0,20408 | 362 | 0,089138 | -0,70503 | 462 | 0,097275 | -0,50634 |
| 63 | 0,192897 | -0,51698 | 163 | 0,123662 | -0,52699 | 263 | 0,129316 | -0,53036 | 363 | 0,234924 | -0,22464 | 463 | 0,085945 | -0,42923 |
| 64 | 0,204242 | -0,35765 | 164 | 0,058355 | -0,70505 | 264 | 0,076759 | -0,54555 | 364 | 0,174614 | -0,28843 | 464 | 0,147832 | -0,46643 |
| 65 | 0,137692 | -0,39933 | 165 | 0,197115 | -0,34759 | 265 | 0,07721 | -0,80881 | 365 | 0,45764 | 0,101286 | 465 | 0,094803 | -0,47872 |
| 66 | 0,056026 | -0,60598 | 166 | 0,194943 | -0,2461 | 266 | 0,068159 | -0,4978 | 366 | 0,268694 | -0,22874 | 466 | 0,098759 | -0,50781 |
| 67 | 0,109849 | -0,46591 | 167 | 0,126274 | -0,41425 | 267 | 0,210844 | -0,30953 | 367 | 0,138057 | -0,37691 | 467 | 0,176952 | -0,40999 |
| 68 | 0,110277 | -0,59911 | 168 | 0,335667 | -0,15603 | 268 | 0,292989 | -0,20042 | 368 | 0,094258 | -0,60526 | 468 | 0,234624 | -0,14334 |
| 69 | 0,108426 | -0,64783 | 169 | 0,081504 | -0,55954 | 269 | 0,242343 | -0,27567 | 369 | 0,254072 | -0,38547 | 469 | 0,146454 | -0,41421 |
| 70 | 0,108844 | -0,45809 | 170 | 0,475321 | 0,197237 | 270 | 0,162976 | -0,49948 | 370 | 0,291453 | -0,28025 | 470 | 0,160801 | -0,35562 |
| 71 | 0,390902 | 0,077298 | 171 | 0,337454 | -0,11024 | 271 | 0,117591 | -0,36901 | 371 | 0,238078 | -0,32583 | 471 | 0,237269 | -0,2396 |
| 72 | 0,134219 | -0,63587 | 172 | 0,070753 | -0,64268 | 272 | 0,159264 | -0,46144 | 372 | 0,228637 | -0,24975 | 472 | 0,166095 | -0,47572 |
| 73 | 0,084473 | -0,7457 | 173 | 0,22388 | -0,44027 | 273 | 0,240172 | -0,32487 | 373 | 0,040967 | -0,66388 | 473 | 0,420425 | 0,082079 |
| 74 | 0,165809 | -0,24259 | 174 | 0,177706 | -0,2713 | 274 | 0,132401 | -0,49613 | 374 | 0,188424 | -0,42635 | 474 | 0,120932 | -0,55438 |
| 75 | 0,196376 | -0,68292 | 175 | 0,291496 | -0,15332 | 275 | 0,256369 | -0,27663 | 375 | 0,129237 | -0,60216 | 475 | 0,017854 | -0,80819 |
| 76 | 0,093177 | -0,62862 | 176 | 0,381309 | -0,06354 | 276 | 0,244207 | -0,38478 | 376 | 0,084482 | -0,55502 | 476 | 0,278322 | -0,12326 |
| 77 | 0,216274 | -0,2802 | 177 | 0,234213 | -0,23509 | 277 | 0,186576 | -0,40474 | 377 | 0,242863 | -0,16549 | 477 | 0,307429 | -0,07025 |
| 78 | 0,083535 | -0,46669 | 178 | 0,128083 | -0,34251 | 278 | 0,366665 | -0,03493 | 378 | 0,134007 | -0,29231 | 478 | 0,082803 | -0,56122 |
| 79 | 0,15407 | -0,42035 | 179 | 0,283863 | -0,1352 | 279 | 0,14703 | -0,36842 | 379 | 0,266693 | -0,12232 | 479 | 0,188583 | -0,20451 |
| 80 | 0,239118 | -0,20131 | 180 | 0,118261 | -0,47031 | 280 | 0,116511 | -0,61687 | 380 | 0,232398 | -0,13879 | 480 | 0,299544 | -0,15399 |
| 81 | 0,216358 | -0,42003 | 181 | 0,198885 | -0,27714 | 281 | 0,120233 | -0,43622 | 381 | 0,220416 | -0,50014 | 481 | 0,143312 | -0,47336 |
| 82 | 0,277644 | -0,17994 | 182 | 0,140725 | -0,61491 | 282 | 0,245265 | -0,17124 | 382 | 0,169338 | -0,32348 | 482 | 0,469755 | 0,186675 |
| 83 | 0,158774 | -0,33811 | 183 | 0,294886 | -0,30813 | 283 | 0,448968 | 0,051671 | 383 | 0,270651 | -0,12259 | 483 | 0,324139 | -0,11357 |
| 84 | 0,222347 | -0,23186 | 184 | 0,219852 | -0,35594 | 284 | 0,105695 | -0,57597 | 384 | 0,158531 | -0,31036 | 484 | 0,030249 | -0,65069 |
| 85 | 0,155305 | -0,51755 | 185 | 0,045406 | -0,66604 | 285 | 0,193885 | -0,51207 | 385 | 0,18463 | -0,68795 | 485 | 0,241143 | -0,26065 |
| 86 | 0,063497 | -0,69258 | 186 | 0,129554 | -0,52005 | 286 | 0,061618 | -0,59125 | 386 | 0,12707 | -0,52217 | 486 | 0,100815 | -0,76309 |
| 87 | 0,185013 | -0,33381 | 187 | 0,195718 | -0,52659 | 287 | 0,069718 | -0,47215 | 387 | 0,171409 | -0,39305 | 487 | 0,262625 | -0,1011 |
| 88 | 0,058581 | -0,71913 | 188 | 0,049444 | -0,53502 | 288 | 0,396742 | -0,05025 | 388 | 0,266607 | -0,2069 | 488 | 0,219107 | -0,14974 |
| 89 | 0,113963 | -0,66974 | 189 | 0,08919 | -0,77503 | 289 | 0,215569 | -0,36521 | 389 | 0,269865 | -0,33683 | 489 | 0,22865 | -0,16897 |
| 90 | 0,197076 | -0,34199 | 190 | 0,026445 | -0,73896 | 290 | 0,365295 | -0,13942 | 390 | 0,118299 | -0,52567 | 490 | 0,213386 | -0,28994 |
| 91 | 0,345461 | -0,13315 | 191 | 0,128707 | -0,29949 | 291 | 0,089643 | -0,54554 | 391 | 0,135872 | -0,56657 | 491 | 0,150617 | -0,48262 |
| 92 | 0,286378 | -0,18643 | 192 | 0,261699 | -0,13055 | 292 | 0,196587 | -0,26549 | 392 | 0,118956 | -0,68736 | 492 | 0,098928 | -0,43442 |
| 93 | 0,206054 | -0,53346 | 193 | 0,104161 | -0,63407 | 293 | 0,287073 | -0,15086 | 393 | 0,304869 | -0,10614 | 493 | 0,201859 | -0,54449 |
| 94 | 0,146511 | -0,56793 | 194 | 0,057089 | -0,55711 | 294 | 0,156106 | -0,45402 | 394 | 0,198469 | -0,23366 | 494 | 0,301467 | -0,08788 |
| 95 | 0,285997 | -0,20487 | 195 | 0,048379 | -0,44437 | 295 | 0,034006 | -0,62013 | 395 | 0,226363 | -0,39686 | 495 | 0,12294 | -0,75031 |
| 96 | 0,026022 | -0,47699 | 196 | 0,245995 | -0,33653 | 296 | 0,179944 | -0,44707 | 396 | 0,30339 | -0,05431 | 496 | 0,075651 | -0,55965 |
| 97 | 0,265752 | -0,25271 | 197 | 0,029026 | -0,45327 | 297 | 0,169397 | -0,27072 | 397 | 0,297057 | -0,218 | 497 | 0,16596 | -0,64269 |
| 98 | 0,151427 | -0,32937 | 198 | 0,112521 | -0,46326 | 298 | 0,22434 | -0,30222 | 398 | 0,196 | -0,35357 | 498 | 0,301698 | -0,1521 |
| 99 | 0,206186 | -0,30101 | 199 | 0,28213 | -0,09441 | 299 | 0,401465 | -0,05793 | 399 | 0,040113 | -0,76999 | 499 | 0,312966 | -0,05432 |
| 100 | 0,151304 | -0,38666 | 200 | 0,085621 | -0,84947 | 300 | 0,263265 | -0,28646 | 400 | 0,055977 | -0,68588 | 500 | 0,291215 | -0,1278 |
